# Breaking the Iron Homeostasis: A “Trojan Horse” Self-Assembled Nanodrug Sensitizes Homologous Recombination Proficient Ovarian Cancer Cells to PARP Inhibition

**DOI:** 10.1021/acsnano.2c04956

**Published:** 2022-08-03

**Authors:** Yangyang Li, Yixuan Cen, Yifeng Fang, Sangsang Tang, Sen Li, Yan Ren, Hongbo Zhang, Weiguo Lu, Junfen Xu

**Affiliations:** † Women’s Reproductive Health Laboratory of Zhejiang Province, Women’s Hospital, Zhejiang University School of Medicine, Hangzhou 310006, Zhejiang, China; ‡ Department of General Surgery, Sir Run Run Shaw Hospital, Zhejiang University School of Medicine, Hangzhou 310016, Zhejiang, China; ∥ Pharmaceutical Sciences Laboratory, Åbo Akademi University, Turku FI-20520, Finland; ¶ Turku Bioscience Centre, University of Turku and Åbo Akademi University, Turku FI-20520, Finland; △ Department of Gynecologic Oncology, Women’s Hospital, Zhejiang University School of Medicine, Hangzhou 310006, ZhejiangChina; ⊥ Cancer Center, Zhejiang University, Hangzhou 310058, Zhejiang, China

**Keywords:** HRR-proficient ovarian cancer, PARP inhibitor, self-assembled olaparib-Ga nanodrug, restrain the tumor growth, negligible toxicity

## Abstract

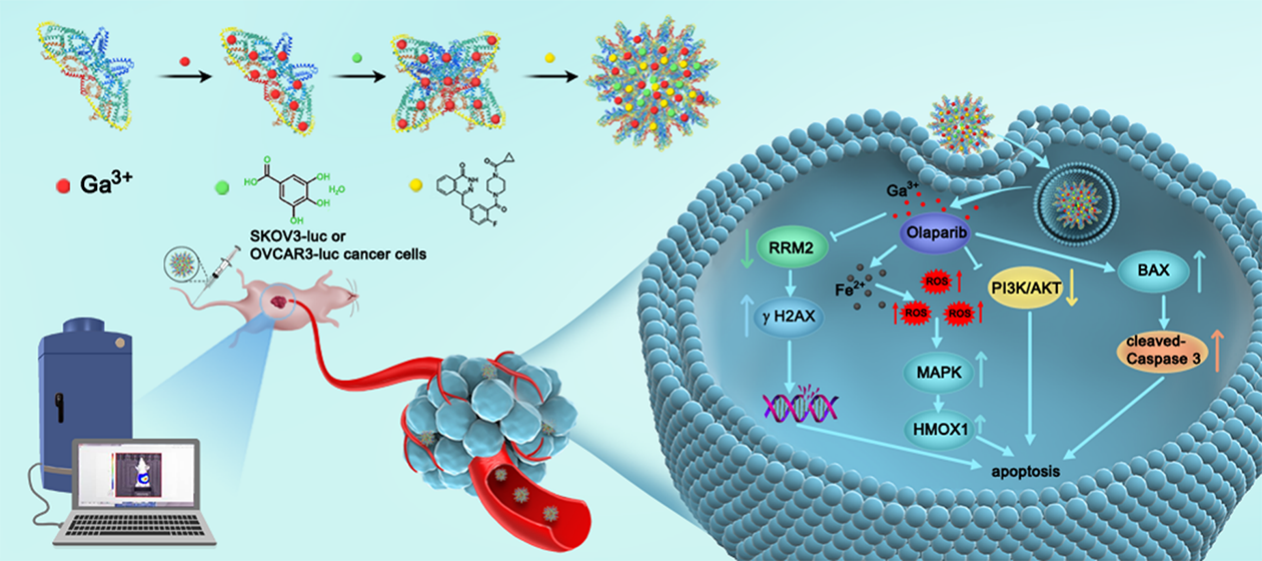

Poly(adenosine diphosphate-ribose) polymerase (PARP) inhibitors are used in ovarian cancer treatment and have greatly improved the survival rates for homologous recombination repair (HRR)-deficient patients. However, their therapeutic efficacy is limited in HRR-proficient ovarian cancer. Thus, sensitizing HRR-proficient ovarian cancer cells to PARP inhibitors is important in clinical practice. Here, a nanodrug, olaparib-Ga, was designed using self-assembly of the PARP inhibitor olaparib into bovine serum albumin through gallic acid gallium(III) coordination *via* a convenient and green synthetic method. Compared with olaparib, olaparib-Ga featured an ultrasmall size of 7 nm and led to increased suppression of cell viability, induction of DNA damage, and enhanced cell apoptosis in the SKOV3 and OVCAR3 HRR-proficient ovarian cancer cells *in vitro*. Further experiments indicated that the olaparib-Ga nanodrug could suppress RRM2 expression, activate the Fe^2+^/ROS/MAPK pathway and HMOX1 signaling, inhibit the PI3K/AKT signaling pathway, and enhance the expression of cleaved-caspase 3 and BAX protein. This, in turn, led to increased cell apoptosis in HRR-proficient ovarian cancer cells. Moreover, olaparib-Ga effectively restrained SKOV3 and OVCAR3 tumor growth and exhibited negligible toxicity *in vivo*. In conclusion, we propose that olaparib-Ga can act as a promising nanodrug for the treatment of HRR-proficient ovarian cancer.

## Introduction

Ovarian cancer is one of the most common malignancies of the female reproductive system and has the highest mortality rate among gynecological cancers.^1−3^ Although surgery and adjuvant chemotherapy are possible therapeutic options, the 5-year survival rate of advanced ovarian cancer remains less than 30% and has not improved in recent years. Recently, the approval of pharmacological poly(adenosine diphosphate-ribose)-ribose polymerase (PARP) inhibitors for clinical use has greatly improved the treatment outcomes for ovarian cancer patients with homologous recombination repair (HRR) deficiency.^4−6^ However, the mutation rate of BRCA1/2 genes in ovarian cancer is relatively low and PARP inhibitors have limited therapeutic efficacy in HRR-proficient cases, which account for half of all ovarian cancer cases.^7^

DNA repair mechanisms are divided into two types, single-strand repair and double-strand repair, and the main enzymes responsible for these two kinds of repair processes are PARP and BRCA, respectively.^8,9^ When BRCA mutations occur in HRR-deficient ovarian cancer, double-strand repair is insufficient.^8,10^ Therefore, when a PARP inhibitor is used to block the single-strand repair pathway,^5,11^ the genetic errors that occur during cell proliferation cannot be repaired.^12^ These cumulative genetic errors result in heavy disruption of DNA functionality, thereby causing tumor cell death. Thus, PARP inhibitors can rapidly induce synthetic lethality, particularly in cells with HRR deficiency. Many clinical trials have demonstrated improved survival rates in ovarian cancer following PARP inhibitor maintenance therapy.^13^ Thus, the National Comprehensive Cancer Network (NCCN) Guideline recommended PARP inhibitors as maintenance therapeutic agents for newly diagnosed and platinum-sensitive recurrent ovarian cancer. However, the use of PARP inhibitors for HRR-proficient ovarian cancer is not as effective as in HRR-deficient types.^6,7^ Indeed, the mutation rate of BRCA genes in ovarian cancer is relatively low, and HRR-proficient ovarian cancer has inherent resistance to DNA damage and apoptosis, which limits the clinical benefit of PARP inhibitors. Therefore, the development of sensitization approaches for PARP inhibitor treatment or alternative combination strategies that can increase sensitivity to PARP inhibitors in HRR-proficient ovarian cancer is urgently needed.

Recently, gallium nitrate, which is approved by the U.S. Food and Drug Agency, has emerged as an alternative anticancer drug, due to its benefits as a combination agent for antitumor therapies.^14,15^ Previous studies have demonstrated that tumor cells have a higher requirement for iron compared with normal cells, which may be attributed to their fast proliferation and to their survival being dependent on the ferric cation. Owing to the very similar chemical properties of iron and gallium, the biological systems cannot distinguish gallium(III) from iron(III).^16^ However, these two cations also differ in their ability to mediate the redox reactions under physiological conditions.^17^ Iron can easily be converted to oxidized states, which is required for the enzymatic activity of ribonucleotide reductase.^18^ The main function of this enzyme is the conversion of ribonucleotides into deoxyribonucleotides (the key building blocks of DNA synthesis).^19^ By contrast, gallium is redox-inactive and can compete with iron when taken up by cells, which disturbs iron homeostasis. In cancer cells, gallium can be utilized to target iron sites, leading to upregulation or hyperactivation of ribonucleotide reductase, thereby inhibiting the DNA synthesis and causing tumor cell death.^20,21^ Therefore, it is important to determine whether disturbing DNA synthesis with gallium(III) can enhance PARP inhibition-mediated tumor suppression, especially in HRR-proficient ovarian cancer patients. Nevertheless, the gallium(III) ionic form may be present in the form of gallium hydroxide salt under physiological conditions, which possesses poor solubility and bioavailability. Moreover, in humans, direct exposure to free gallium(III) ions can induce adverse effects such as nephrotoxicity and renal acidosis.^22,23^ Therefore, developing a drug formulation that can co-deliver gallium(III) and PARP inhibitors simultaneously may improve the treatment outcomes of HRR-proficient ovarian cancer.

**Figure 1:**
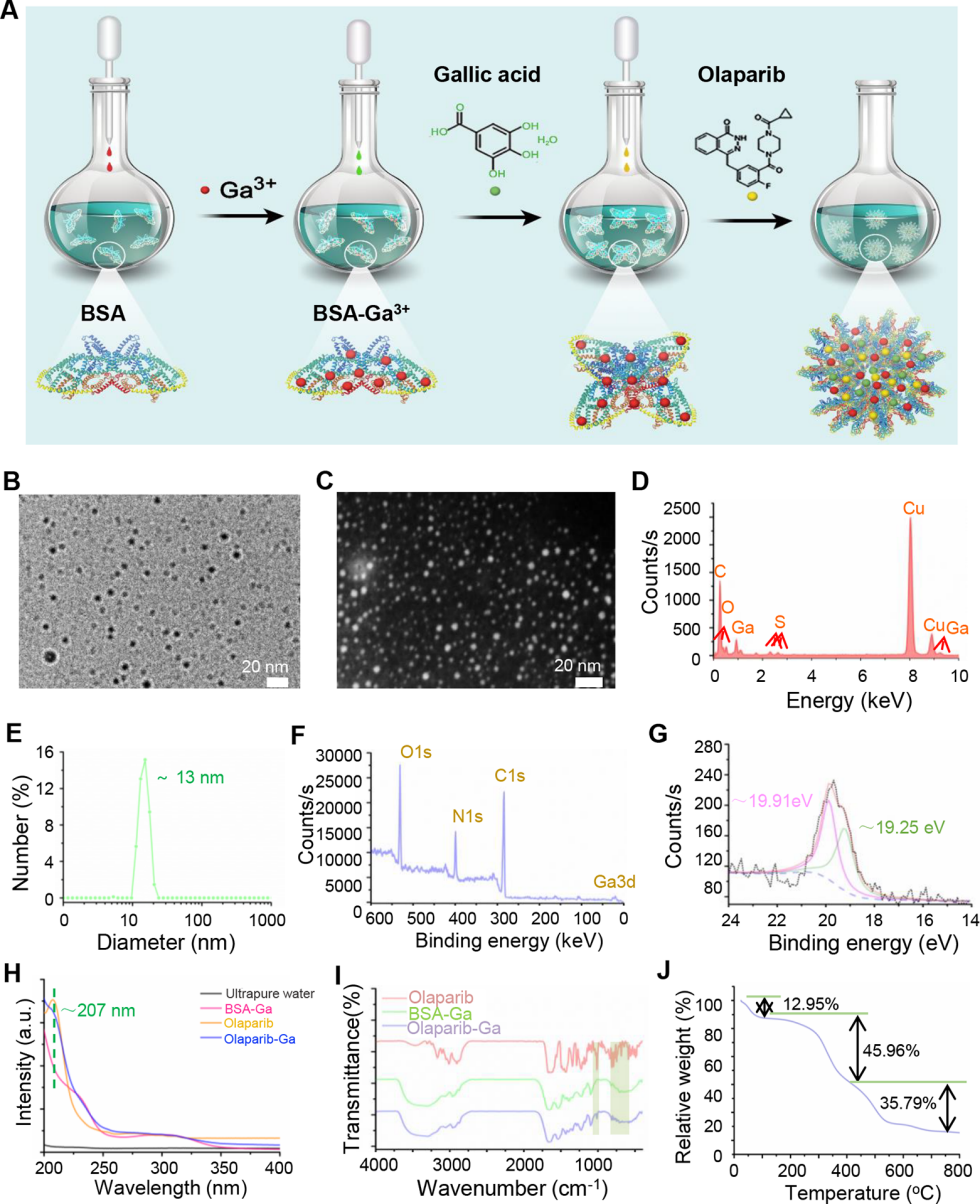
Synthesis and characterization of the olaparib-Ga nanodrug. (A) Workflow of the synthesis of olaparib-Ga. (B and C) Representative TEM micrographs. Scale bar, 20 nm. (D) Representative EDX pattern images of olaparib-Ga. (E) Dynamic light scattering data of olaparib-Ga in aqueous solution. (F and G) XPS spectrum of olaparib-Ga and spectra of Ga 3d orbitals of olaparib-Ga for the binding energies together with the fitting curves. (H and I) FT-IR and vis–UV spectra of olaparib-Ga. (J) Thermogravimetric (TG) analysis of olaparib-Ga under an air atmosphere.

Here, a straightforward BSA-assisted biomineralization method was used for the preparation of a biocompatible “all-in-one” olaparib-Ga nanodrug (Figure 1A). First, a BSA-Ga^3+^ complex was prepared. The BSA molecule contains several N-terminal amine groups and cysteine residues, which can coordinate with gallium metal ions (Ga^3+^).^24,25^ Second, gallic acid (GA) was mixed with the BSA-Ga^3+^ complex to synthesize a BSA-stabilized GA-Ga^3+^ by chelating the phenolic groups and the carboxylic groups in the GA molecule with Ga^3+^. During this process, GA served as a ligand to react with Ga^3+^ to form a stable metal–organic coordination nanoformulation. It has also been reported that GA could exhibit certain antitumor activities.^26,27^ Finally, olaparib, a universal PARP inhibitor, was incorporated into the BSA-Ga^3+^ complex *via* the hydrophobic effect to form the olaparib-Ga nanodrug through self-assembly.^28−31^ The anticancer effects of the olaparib-Ga nanodrug were examined in SKOV3 and OVCAR3 human HRR-proficient ovarian cancer cells. Subsequently, the effects of olaparib-Ga on suppression of RRM2 expression, activation of Fe^2+^/reactive oxygen species (ROS)/MAPK and HMOX1 signaling, inhibition of the PI3K/AKT signaling pathway, and cleaved-caspase 3 and BAX protein upregulation were evaluated in order to compare the tumor-suppressive role of olaparib-Ga with pure olaparib. Moreover, *in vivo* experiments were carried out to demonstrate the antitumor effects and the biocompatibility of the nanodrug. Our findings uncover the application of the newly developed olaparib-Ga nanodrug in the treatment of HRR-proficient ovarian cancer.

## Results and Discussion

### Synthesis and Characterization of the Olaparib-Ga Self-Assembled Nanodrug

The olaparib-Ga self-assembled nanodrug was synthesized using a simple method based on a previous study (Figure 1A).^32^ Specifically, bovine serum albumin (BSA) was first added as the surfactant to form the BSA-Ga^3+^ complex. Subsequently, GA was mixed with the BSA-Ga^3+^ complex. The phenolic groups and carboxylic groups in GA molecules were able to react with Ga^3+^ to form a stable GA-Ga^3+^ by the formation of phenolate carboxylate group-Ga^3+^ coordination bonds. Then, the BSA protein can be used for connecting with hydrophobic drugs (olaparib). Moreover, the BSA protein can serve as a stabilizer to form the nanodrug. Ultimately, the olaparib-Ga self-assembled nanodrug was formed by adding the olaparib molecules in dimethyl sulfoxide solution to the above complex solution. The morphology and size of the olaparib-Ga nanodrug was determined by transmission electron microscopy (TEM) and dynamic light scattering (DLS), respectively. As shown in Figure 1B and C, the olaparib-Ga self-assembled nanodrug consisted of spherical monodispersed nanoparticles with an average size of ∼7 nm. Furthermore, the energy-dispersive X-ray spectroscopy (EDS) elemental spectrum demonstrated the presence of the C, O, S, and Ga elements (Figure 1D). From the elemental mapping pattern of the olaparib-Ga nanodrug, N element was detected, which belonged to the BSA protein. F element was attributed to the olaparib molecule (Figure S1A and B). In addition, the hydrodynamic diameter of the olaparib-Ga nanodrug was ∼13 nm, and the slightly increased hydrodynamic size compared with the TEM size may be due to the certain aggregation in aqueous solution (Figure 1E). Moreover, X-ray photoelectron spectroscopy (XPS) measurements were used to analyze element composition and validate the valence of the Ga elements (Figure 1F and G). The characteristic peaks of O 1s, N 1s, C 1s, and Ga 3d were observed. The two binding energy peaks at ∼19.91 and ∼19.25 eV may belong to the Ga^3+^ and Ga^δ+^ types, respectively (Figure 1G).^33^ The vis–UV spectra of the olaparib molecule showed a distinct peak at ∼207 nm, which is similar to that of the olaparib-Ga nanodrug (Figure 1H). The FT-IR spectra of different groups are shown in Figure 1I. The characteristic peaks of the olaparib molecule around the 1285 and 1015 cm^–1^ regions may be attributed to the C–O, C–N, and C–F stretching vibration absorptions. The absorption bands around 805 cm^–1^ may belong to the C=C stretching vibration absorption in the benzene ring structure of the olaparib molecule. The prominent peaks at 1650 cm cm^–1^ (amide I and mainly C=O stretching vibrations) and a broad absorption region around 3500 cm cm^–1^ (O–H stretch) were observed in olaparib-Ga.^32,34^ Moreover, the characteristic region of BSA-amide band vibrations around the 1650 and 1540 cm^–1^ regions indicated the BSA protein was within the olaparib-Ga nanodrug matrix.^35^ The concentrations of Ga^3+^ in the olaparib-Ga nanodrug was calculated by inductively coupled plasma mass spectroscopy (ICP-MS), and the concentration of Ga^3+^ was ∼250 μg/mL. The loading efficiency of gallic acid and olaparib in the nanodrug was quantified and calculated by the UV–vis spectroscopy method (Figure S1C and D). The loading efficiency of olaparib was 12.4 ± 2.26%, and that of gallic acid was 61.2 ± 1.53%. Subsequently, we investigated the release kinetics of olaparib, the gallic acid molecule, and Ga^3+^ when immersing the nanodrug in different pH conditions (Figure S2). As shown in Figure S2A, as the pH value decreases from 7.4 to 5.8, the olaparib drug released faster and exhibited a pH-responsive release behavior. The gallic acid molecule and Ga^3+^ also demonstrated similar release kinetic characteristics (Figure S2B and C). Finally, the thermogravimetric analysis (TG) of olaparib-Ga further confirmed the successful conjugation of the inorganic Ga^3+^ component with the polymer compound (Figure 1J). With increasing the temperature to around 120 °C, a ∼12.95% weight loss was detected, which was due to the evaporation of adsorbed water. The ∼45.96% weight loss during the second attenuation region between ∼120 and ∼410 °C was mainly due to the decomposition of small molecules in the nanodrug. The ∼35.79% weight loss during the temperature increase from the ∼410 °C to ∼800 °C was mainly a result of the decomposition of macromolecular proteins. Collectively, these results demonstrated the successful preparation of the olaparib-Ga nanodrug.

### Biodistribution of Olaparib-Ga *in Vivo*

The biodistribution of olaparib-Ga was evaluated using an HRR-proficient ovarian cancer SKOV3-derived tumor xenograft model (Figure S3A). The luciferase-expressing SKOV3 (SKOV3-luc) cells were intraperitoneally injected into the mice (*n* = 6). Seven days after transplantation, the bioluminescence intensity was determined (Figure S3B). The mice were then randomized into two groups (*n* = 3 each group). The SKOV3 tumor-bearing mice were intravenously injected with free IR780 or IR780/olaparib-Ga. The fluorescence signal images were captured using an *in vivo* imaging system (IVIS) before injection (set as 0 h) and at different time points after injection, including 0.5, 1, 2, 6, and 24 h postinjection (Figure S3C). We sacrificed one mouse from each group to detach tumors and major organs including the heart, liver, spleen, lung, kidney, and brain for fluorescence signal evaluation at 6 h postinjection. The tissue distribution of IR780/olaparib-Ga showed a higher signal accumulation in tumors than the free IR780 counterpart. The IR780/olaparib-Ga also had a higher retention in tumor tissues than in major organs (Figure S3D). The remaining four mice were evaluated at 24 h postinjection. We found that the fluorescence signal of pure IR780 was almost undetectable at this time point. This may indicate that the free IR780 has already been eliminated. In the IR780/olaparib-Ga group, the fluorescence signal was also much weaker than that at 6 h postinjection, but it was mainly distributed in the xenograft tumors (Figure S3C and E).

### Antitumor Performance of the Olaparib-Ga Nanodrug *in Vitro*

**Figure 2:**
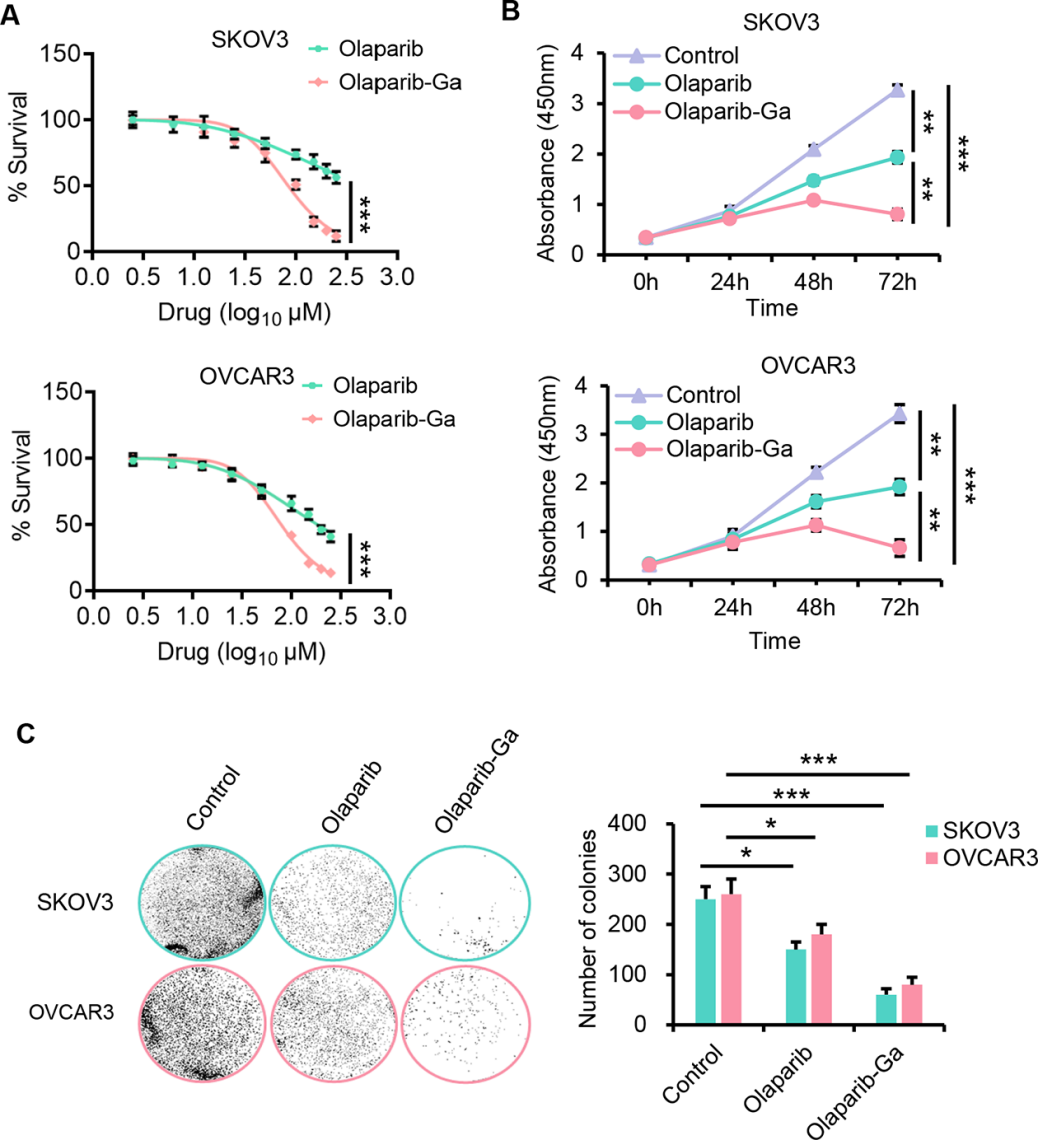
Olaparib-Ga nanodrug inhibits the growth of HRR-proficient SKOV3 and OVCAR3 ovarian cancer cells. (A) Dose–response curves of the cell viability of SKOV3 and OVCAR3 cancer cells following treatment with different concentrations of olaparib-Ga or olaparib obtained using CCK-8 assays at 48 h. The data are presented as mean ± SD. IC_50_ was calculated by Graph Pad Prism 9.0. The calculation of the IC_50_ of the olaparib-Ga nanodrug was based on the concentration of olaparib. (B) SKOV3 and OVCAR cells were treated with olaparib or olaparib-Ga using the IC_50_ of the olaparib-Ga nanodrug, and cell viability was measured using time-lapse imaging for 72 h in the continued presence of the drugs in a CCK-8 assay. (C) SKOV3 and OVCAR3 cells were treated with olaparib or olaparib-Ga for 10 days, and colony formation assays were analyzed. Representative images and quantization of the number of colonies are shown. *, *P* < 0.05; **, *P* < 0.01; ***, *P* < 0.001.

To test the anticancer activity of olaparib-Ga against HRR-proficient ovarian cancer, the HRR-proficient SKOV3 and OVCAR3 ovarian cancer cell lines were used.^36^ Both cell lines exhibited similar dose-dependent tumor cell killing abilities following treatment with different concentrations of olaparib or olaparib-Ga for 48 h, as shown in Figure 2A. Interestingly, the half-maximal inhibitory concentration (IC_50_) of olaparib-Ga was much lower than that of olaparib in both cell lines at 48 h. Moreover, olaparib-Ga also exhibited markedly more potent antitumor cell activity against the two cell lines in a time-dependent manner (Figure 2B). Moreover, colony formation assays were conducted to evaluate the effect of the drugs on cancer cell proliferation. As shown in Figure 2C, olaparib-Ga treatment was more potent in inhibiting colony formation ability compared with the control or olaparib group. The improved effects of the olaparib-Ga nanodrug may be due to the presence of gallium(III), which may have a synergistic effect with olaparib, thus increasing the sensitivity of HRR-proficient ovarian cancer cells to this drug.

**Figure 3:**
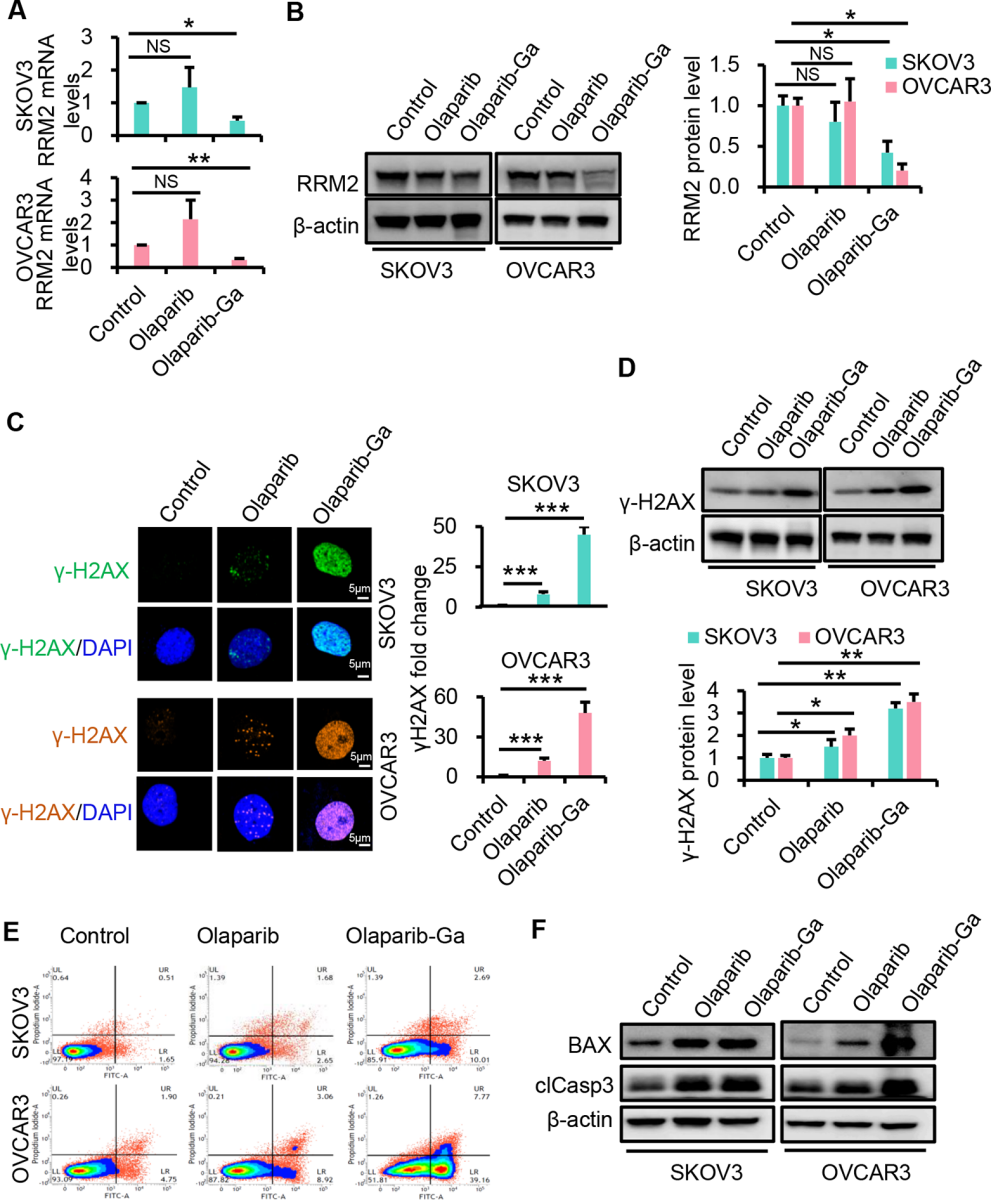
Olaparib-Ga nanodrug disturbs DNA synthesis, induces damage, and promotes apoptosis in SKOV3 and OVCAR3 cells. (A) RT-qPCR analysis of RRM2 mRNA expression after treatment with olaparib or olaparib-Ga for 48 h. (B) Relative expression of RRM2 protein in SKOV3 and OVCAR3 cells treated with olaparib or olaparib-Ga examined by Western blotting. The protein levels of RRM2 were semiquantified. (C) SKOV3 and OVCAR3 cells after treatment with olaparib or olaparib-Ga for 48 h. Co-IF for γH2AX and DAPI in the nucleus was performed. Scale bar, 5 μm. The γH2AX levels were quantified in each group. (D) γH2AX protein levels examined using Western blotting analysis. β-Actin was used as a control. The γH2AX levels were quantified in each group. (E) SKOV3 and OVCAR3 cells after treatment with olaparib or olaparib-Ga for 48 h. Apoptosis was analyzed by flow cytometry using annexin V and PI staining. (F) Expression of the pro-apoptotic proteins BAX and cleaved-caspase 3 (clCasp 3) in SKOV3 and OVCAR3 cells after treatment with olaparib or olaparib-Ga. *, *P* < 0.05; **, *P* < 0.01; ***, *P* < 0.001; NS, not significant.

Iron is essential for the fast proliferation and survival of cancer cells, as it is required for the activity of the R2 subunit of ribonucleotide reductase, which is responsible for deoxyribonucleotide synthesis, a rate-limiting step in DNA synthesis.^37^ Gallium can be used to disrupt cancer cell iron metabolism. Thus, the iron-dependent R2 subunit of ribonucleotide reductase may be inhibited by gallium(III).^38^ Here, the levels of ribonucleotide reductase regulatory subunit M2 (RRM2) were examined using RT-qPCR and Western blotting in SKOV3 and OVCAR3 cells following olaparib-Ga treatment. As shown in Figures 3A and B, the RRM2 mRNA levels were downregulated after olaparib-Ga treatment compared with olaparib-treated or control cells. Consistent with the mRNA results, the RRM2 protein expression levels also significantly decreased after olaparib-Ga nanodrug treatment. Therefore, the olaparib-Ga nanodrug may restrain DNA synthesis *via* RRM2, which is possibly mediated by gallium(III).

PARP inhibitors inhibit DNA repair and induce DNA damage in HRR-deficient ovarian cells.^9,39^ However, HRR-proficient ovarian cancer cells exhibit inherent resistance to DNA damage and apoptosis, limiting the clinical benefit of PARP inhibitors.^7^ Here, the DNA damage inhibition by the olaparib-Ga nanodrug was examined in HRR-proficient SKOV3 and OVCAR3 cancer cells. After 48 h of incubation with olaparib-Ga, the levels of γH2AX (Ser139), a surrogate marker for DNA damage, were evaluated. As shown in Figure 3C, the fluorescence intensity of γH2AX significantly increased following olaparib-Ga treatment in both cell lines, and increased levels of γH2AX protein expression were also observed by Western blotting (Figure 3D). These results indicated increased DNA damage in the olaparib-Ga treatment group, which may be attributed to disruption of RRM2 by gallium(III). Subsequently, apoptosis assays were conducted. As shown in Figures 3E and S4A, olaparib only slightly induced apoptosis in SKOV3 and OVCAR3 cells, whereas olaparib-Ga significantly induced apoptosis in these HRR-proficient cells, with an approximately 5-fold increase compared with olaparib treatment in both cell lines. Furthermore, the expression of the apoptosis-related proteins, BAX and cleaved-caspase 3, was examined using Western blotting (Figures 3F and S4B). Both the olaparib-Ga and the olaparib treatment groups upregulated the expression levels of BAX and cleaved-caspase 3 protein compared with the control group. However, the protein levels of BAX and cleaved-caspase 3 were significantly higher in olaparib-Ga-treated cells compared with those treated with olaparib. Therefore, gallium(III) has a synergistic effect on DNA damage with olaparib and thus promotes the apoptosis of HRR-proficient ovarian cancer cells.

### Mechanical Roles of the Synergistic Antitumor Effects of Olaparib-Ga

**Figure 4:**
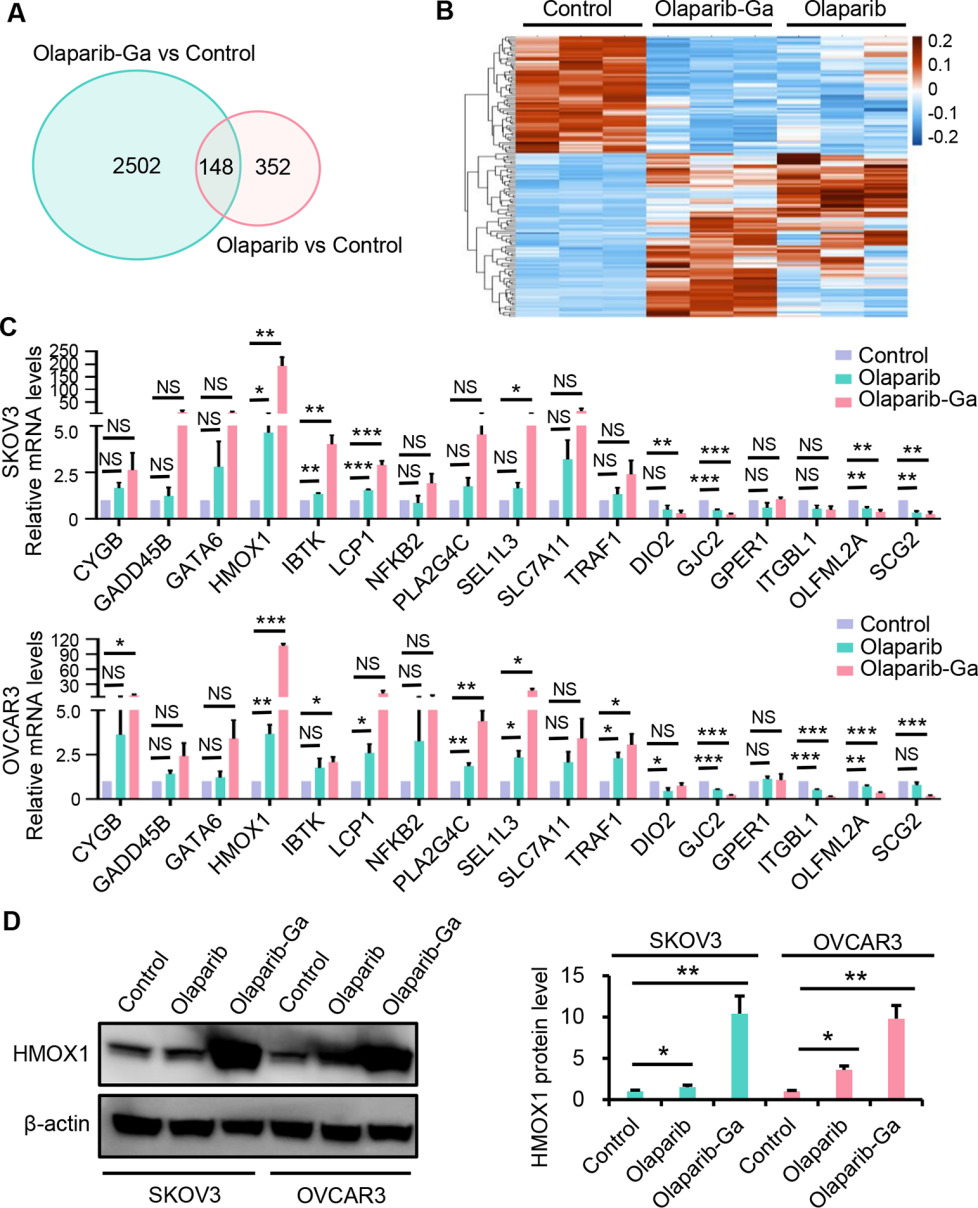
RNA-seq analysis of OVCAR3 cells treated with olaparib or the olaparib-Ga nanodrug. OVCAR3 cells were treated with olaparib or olaparib-Ga for 48 h for RNA-seq analysis. (A) Venn diagram showing the differentially expressed genes that were shared between the olaparib *vs* control group and the olaparib-Ga *vs* control group. (B) Heatmap showing the 148 overlapping, differentially expressed genes in the control, olaparib, and olaparib-Ga-treated groups. (C) RT-qPCR validation of the 17 genes selected from those 148 genes in SKOV3 and OVCAR3 cells treated with olaparib or olaparib-Ga for 48 h. (D) SKOV3 and OVCAR3 cells were treated with olaparib or olaparib-Ga, and the protein expression of HMOX1 protein was evaluated using Western blotting. The data are shown as mean + SD of three independent experiments and are normalized to the control group. *, *P* < 0.05; **, *P* < 0.01; ***, *P* < 0.001; NS, not significant.

To understand the detailed mechanisms underlying the observed antitumor effects of the olaparib-Ga nanodrug, we conducted a RNA-seq analysis of OVCAR3 cells treated with olaparib, olaparib-Ga, or control reagents. A total of 500 differentially expressed genes were identified for olaparib-treated cells and 2650 for olaparib-Ga-treated cells relative to control cells. Among these, 148 genes were differentially expressed in both data sets, with 87 being upregulated and 61 being downregulated in both groups (Figure 4A and B, Table S1). Moreover, Path-Act-Network analysis was performed to identify the pathways enriched in both the olaparib and olaparib-Ga groups relative to the control group. The results indicated that the “MAPK signaling pathway”, “NF-kappa B signaling pathway”, “HIF-1 signaling pathway”, “FOXO signaling pathway”, and “VEGF signaling pathway” were enriched (Figure S5). To verify the differentially expressed genes identified by RNA-seq, 17 genes from the 148 genes in SKOV3 and OVCAR3 cells were examined using RT-qPCR (Figure 4C). The 17 genes were chosen on the basis of the top network analysis and the function of these genes, as well as their altered gene expression levels. Although these genes demonstrated a similar expression pattern to that seen in RNA-seq in response to olaparib or olaparib-Ga in both SKOV3 and OVCAR3 cell lines, these results were not statistically significant. Nevertheless, the increased expression of HMOX1 and the decreased expression of GJC2 and OLFML2A were statistically significant in olaparib- and olaparib-Ga-treated SKOV3 and OVCAR3 groups over the control group (Figure 4C). Notably, these changes in gene expression in olaparib-Ga-treated cells were higher than those seen in olaparib-treated cells relative to the control group.

**Figure 5:**
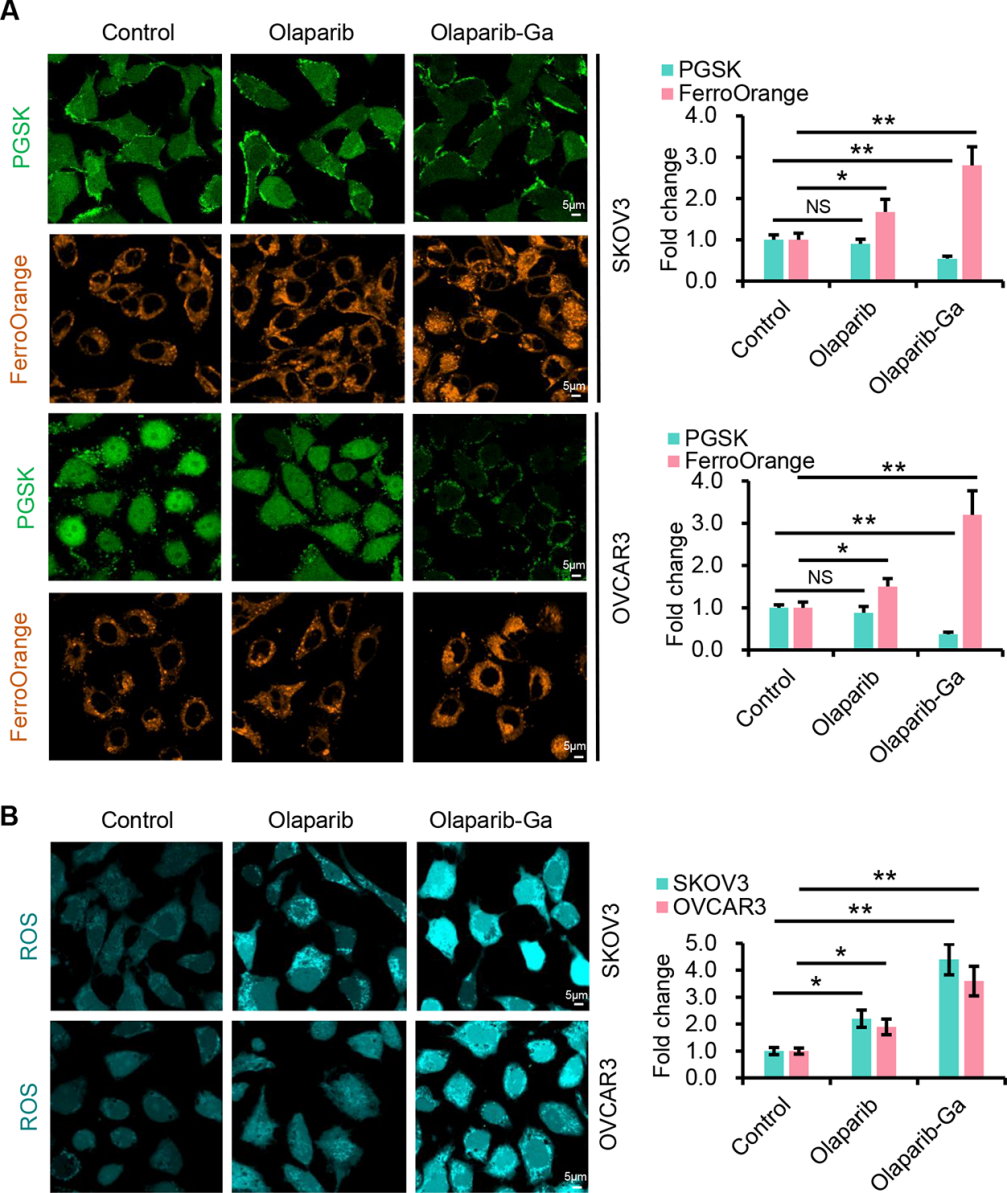
Olaparib-Ga nanodrug enhances Fe^2+^/ROS in SKOV3 and OVCAR3 cells. (A) SKOV3 and OVCAR3 cells after treatment with olaparib or olaparib-Ga nanodrug for 3 h. Intracellular iron was labeled using the fluorescent probe PGSK. Fe^2+^ was detected using FerroOrange. Representative confocal laser scanning microscopy (CLSM) images of PGSK and FerroOrange staining in SKOV3 and OVCAR3 cells. Scale bars, 5 μm. Quantification of the fluorescence intensity of PGSK and FerroOrange is shown on the right. (B) Intracellular ROS generation determined in SKOV3 and OVCAR3 cells after treatment with olaparib or olaparib-Ga for 3 h. Representative CLSM images of ROS staining with the green fluorescence signal confirming intracellular ROS generation. Scale bars, 5 μm. Quantification of the fluorescence intensity is shown on the right. *, *P* < 0.05; **, *P* < 0.01; ***, *P* < 0.001; NS, not significant.

We further confirmed the expression of the HMOX1 protein by Western blotting in SKOV3 and OVCAR3 cells (Figure 4D). Although the expression of HMOX1 protein was only slightly increased in response to olaparib treatment, it was markedly increased in olaparib-Ga-treated cells, compared with olaparib-treated and control cells. A previous study has suggested that the upregulation of HMOX1 may be ascribed to increased levels of ROS.^14^ Thus, enhanced expression of HMOX1 following treatment with olaparib-Ga may be achieved through the MAPK pathway in response to ROS.^14,40^ Therefore, we examined the intracellular iron and ROS levels. The green fluorescence of PGSK was reduced and the levels of the Fe^2+^ indicator of FerroOrange were markedly enhanced after olaparib-Ga nanodrug treatment in both SKOV3 and OVCAR3 cell lines (Figure 5A), indicating increased ferroptosis, which may be caused by endoplasmic reticulum stress.^41^ Next, we sought to determine whether rising Fe^2+^ content would also regulate the accumulation of ROS. As demonstrated in Figure 5B, treatment with the olaparib-Ga nanodrug significantly increased ROS levels compared with the olaparib and control groups, in both SKOV3 and OVCAR3 cells. These findings suggest that the increased HMOX1 gene expression in olaparib-Ga-treated cells could be due to the increased Fe^2+^-induced ROS response.

**Figure 6:**
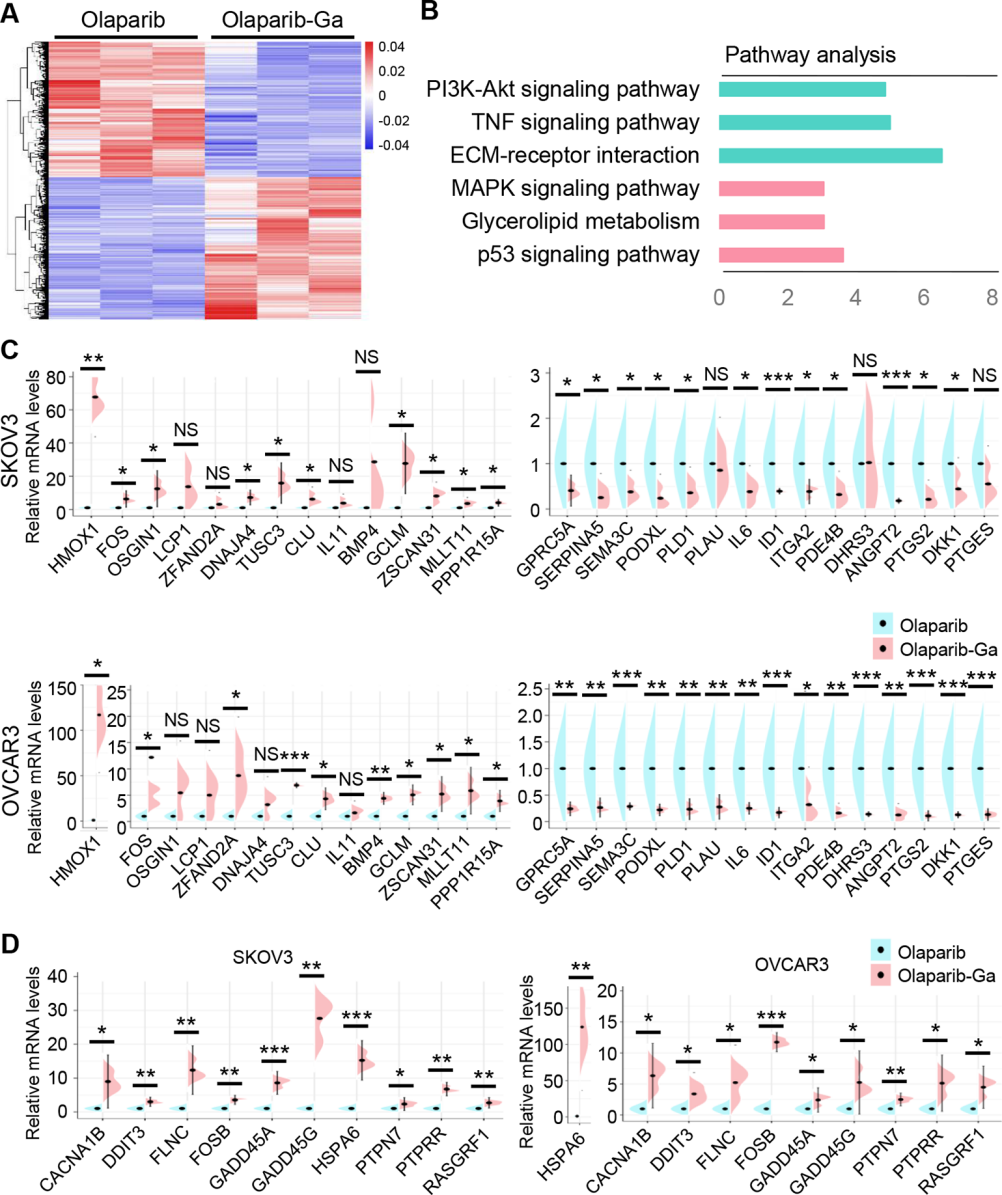
RNA-seq analysis of the differential changes between olaparib-Ga- and olaparib-treated HRR-proficient ovarian cancer cells. (A) Heatmap showing 3109 differentially expressed genes in OVCAR3 cells following treatment with olaparib-Ga or olaparib. (B) Top six most significant KEGG pathways between olaparib-Ga- and olaparib-treated groups. Red, activated; green, inhibited. (C) RT-qPCR validation of the 29 genes in SKOV3 and OVCAR3 cells treated with olaparib or olaparib-Ga for 48 h. (D) RT-qPCR validation of the 10 top genes of the MAPK pathway in SKOV3 and OVCAR3 cells treated with olaparib or olaparib-Ga. *, *P* < 0.05; **, *P* < 0.01; ***, *P* < 0.001; NS, not significant.

In addition, we further investigated whether other biological mechanisms were involved in the improved performance of the olaparib-Ga nanodrug *versus* olaparib. We analyzed the differentially expressed genes from the RNA-seq results. We identified 3109 altered genes in the olaparib-Ga-treated group relative to the olaparib-treated group (Figure 6A). KEGG pathway analysis showed that the “MAPK signaling pathway”, “glycerolipid metabolism”, and “p53 signaling pathway” were significantly activated, while the “PI3K-AKT signaling pathway”, “TNF signaling pathway”, and “ECM–receptor interaction” were significantly inhibited in response to olaparib-Ga treatment as compared with olaparib treatment (Figure 6B). We further selected the 14 top upregulated and 15 most downregulated genes from the RNA-seq data for validation. RT-qPCR results revealed that HMOX1, FOS, TUSC3, CLU, GCLM, ZSCAN31, MLLT11, and PPP1R15A were significantly upregulated and that GPRC5A, SERPINA5, SEMA3C, PODXL, PLD1, IL6, ID1, ITGA2, PDE4B, ANGPT2, PTGS2, and DKK1 were significantly downregulated in the olaparib-Ga-treated group compared with the olaparib-treated group (Figure 6C). It has been reported that the MAPK pathway mediates ROS-induced HMOX1 expression.^42^ Thus, we validated the top 10 differentially expressed genes from the MAPK pathway. As shown in Figure 6D, SKOV3 and OVCAR3 cells treated with olaparib-Ga upregulated the expression of MAPK pathway-associated genes, including CACNA1B, DDIT3, FLNC, FOSB, GADD45A, GADD45G, HSPA6, PTPN7, PTPRR, and RASGRF1, compared with olaparib-treated cancer cells. These results are in agreement with the RNA-seq data and with the findings of previous studies.^14,43^ In addition to the activation of the Fe^2+^/ROS/MAPK pathway and HMOX1 signaling, we further evaluated whether the inhibition of the PI3K/AKT pathway identified in the RNA-seq data could also account for the suppressive roles of olaparib-Ga nanodrug treatment. The results suggested that treatment of SKOV3 and OVCAR3 cells with olaparib-Ga markedly reduced the levels of p-PI3K and p-AKT (Figure S6). In summary, these data demonstrated that olaparib-Ga resulted in RRM2 inhibition and DNA damage, activation of Fe^2+^/ROS/MAPK and HMOX1 signaling, and suppression of the PI3K/AKT pathway in HRR-proficient SKOV3 and OVCAR3 cancer cells. It has been reported that dysregulation of these signaling pathways could contribute to cancer cell apoptosis.^42,44,45^ Altogether, our results indicated that olaparib-Ga could promote cancer cell apoptosis *via* synergy between Ga ions and olaparib.

### *In Vivo* Antitumor Efficiency and Preliminary Evaluation of Biosafety

**Figure 7:**
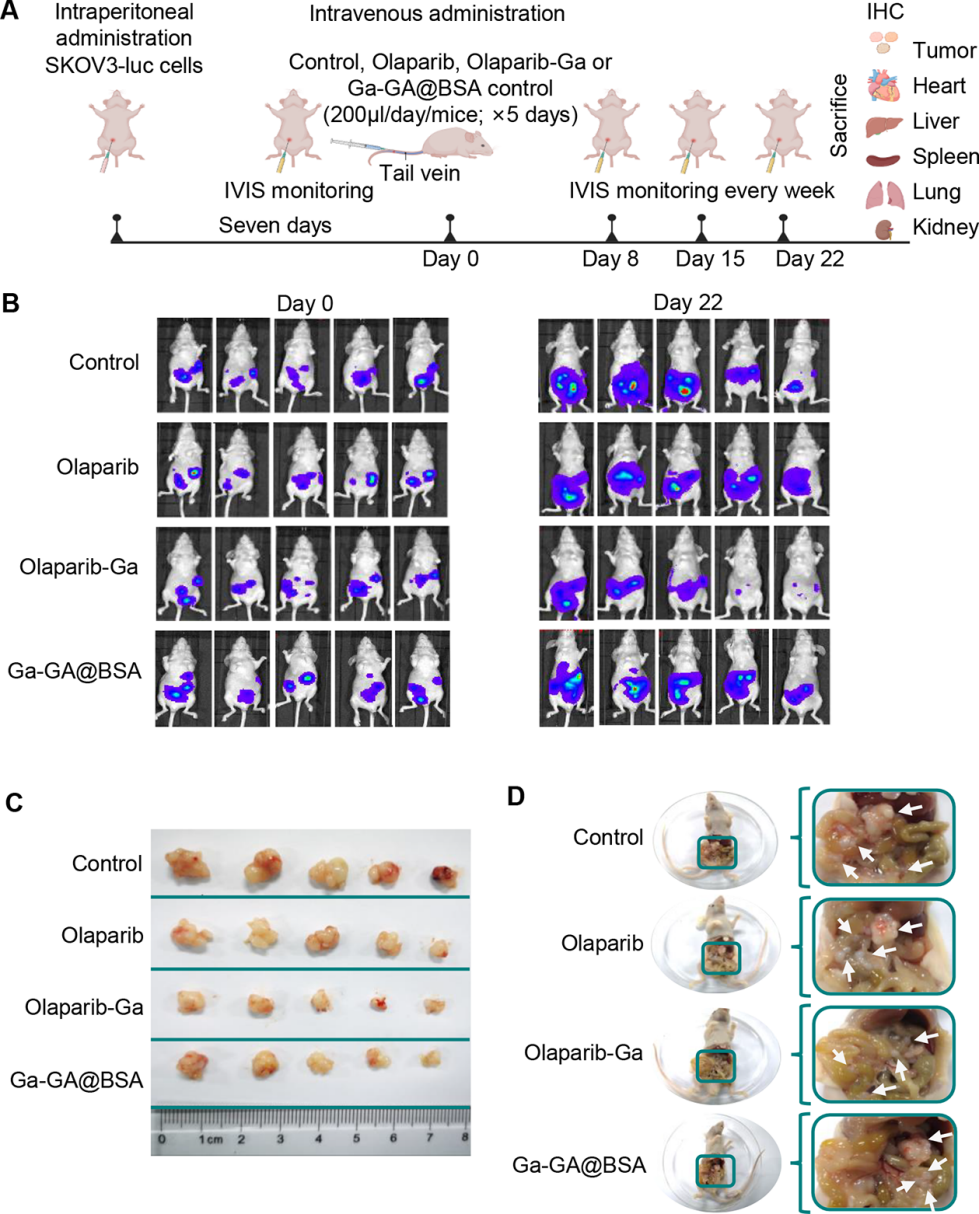
Antitumor effects of the olaparib-Ga nanodrug against SKOV3-derived xenograft tumors. (A) Experimental schematic of olaparib-Ga inhibition of tumor growth in a SKOV3-luc-derived xenograft model. Mice bearing luciferase SKOV3-derived tumors were treated with olaparib, olaparib-Ga, or Ga-GA@BSA control (200 μL/mouse, QD × 5 days; *n* = 5 mice per group). Tumor growth was monitored weekly using bioluminescence imaging in the mice. (B) Representative bioluminescence images of mice bearing SKOV3-derived tumors at day 0 and day 22. (C) Representative images of SKOV3-luc-derived xenograft tumors. (D) Representative images of the SKOV3-luc-derived xenograft tumors in mice.

**Figure 8:**
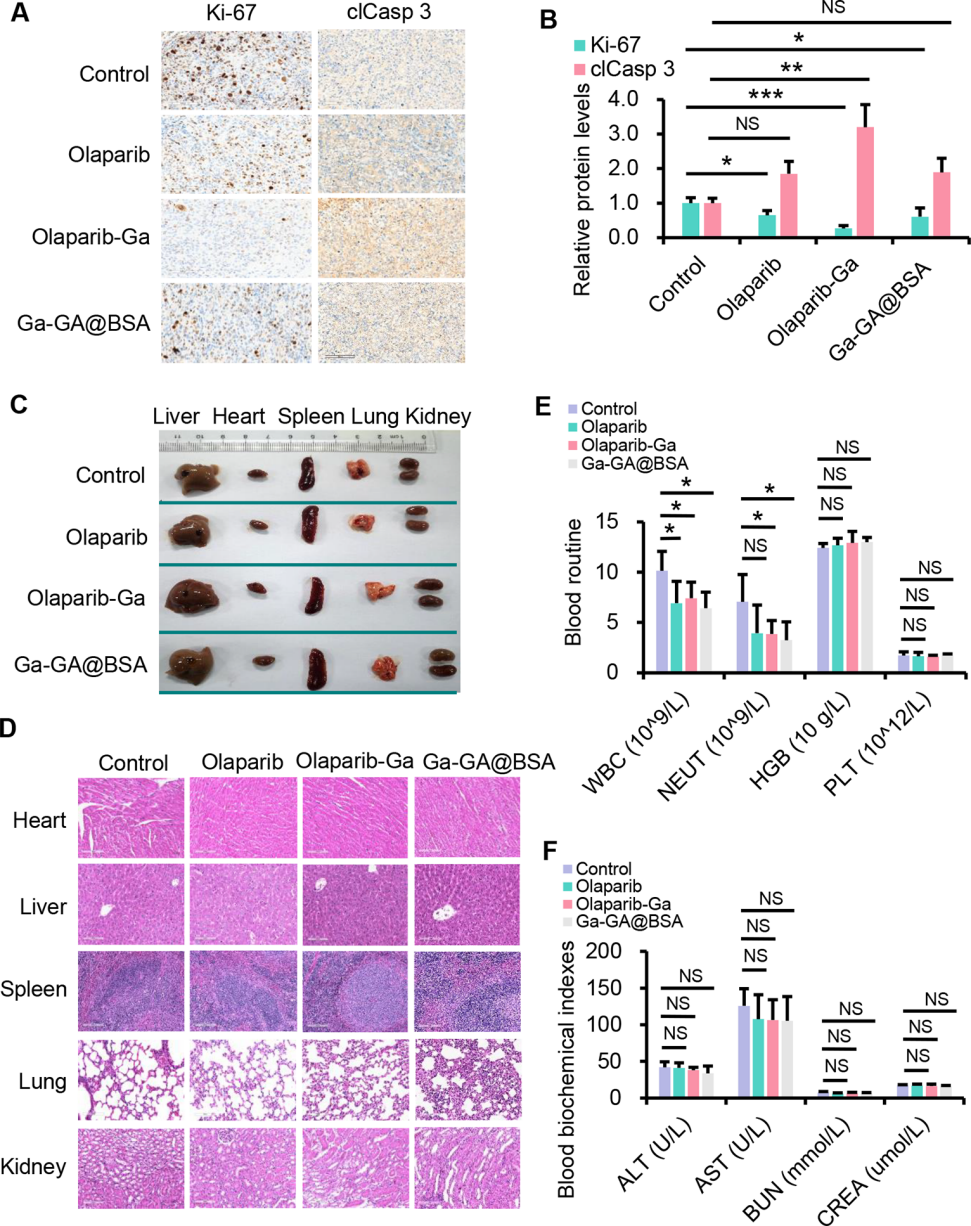
Preliminary toxicity analysis of olaparib-Ga in mice bearing SKOV3-derived xenograft tumors. (A and B) Immunohistochemistry for Ki-67 and clCasp 3 in SKOV3-derived xenograft tumors with olaparib, olaparib-Ga, or Ga-GA@BSA treatment. Relative protein expression is shown as the mean + SD for each treatment group and normalized to the control group. (C) Representative photographs of the heart, liver, spleen, lung, and kidney harvested from mice in different treatment groups following treatment. (D) Representative H&E staining images of these major organs. (E and F) Blood routine and blood biochemistry tests were carried out in the mice. Data are presented as mean + SD. *, *P* < 0.05; **, *P* < 0.01; ***, *P* < 0.001; NS, not significant.

To further support our *in vitro* findings and to confirm the potential clinical implications, SKOV3-derived and OVCAR3-derived tumor xenograft models were established. These luciferase-expressing tumor cells (SKOV3-luc and OVCAR3-luc) were injected into mice intraperitoneally. During the process of model establishment, the luciferase signal was monitored using an IVIS. Subsequently, the *in vivo* efficacy of olaparib-Ga against the growth of HRR-proficient ovarian tumors was investigated. Figure 7A describes the detailed procedure for the animal experiment. Here, mice treated with the same dose of Ga-GA@BSA nanodrug but without olaparib was also used as the control. In the SKOV3-luc model, the treatment began 1 week after the inoculation of SKOV3-luc cells. The mice were divided into four groups and treated with the control, olaparib, olaparib-Ga, or Ga-GA@BSA. The luciferase signal of SKOV3-luc cells was detected using noninvasive bioluminescence imaging for assessing the therapeutic effects. The bioluminescence images from each group (*n* = 5) and the quantification of the bioluminescence intensities at day 0 and day 22 are shown in Figures 7B and S7. There were no differences in bioluminescence intensity between any of the groups at day 0. However, at day 22, the olaparib-Ga-treated group exhibited the lowest bioluminescence intensity, that is, the highest therapeutic effect. In contrast, the olaparib treatment and Ga-GA@BSA treatment induced limited tumor-suppressive effects against the growth of SKOV3 tumors. The mice were then sacrificed, and the tumors from each mouse were obtained and photographed using a digital camera. As expected, the tumors derived from the SKOV3-luc cells treated with olaparib-Ga were much smaller than those treated with olaparib, Ga-GA@BSA, or control reagents, which suggests that olaparib-Ga treatment could significantly inhibit tumor growth (Figure 7C). More importantly, olaparib-Ga treatment also markedly inhibited the metastasis of SKOV3-luc-derived tumors, as evidenced by reduced formation of abdominal tumor nodules in the colon and liver compared with the olaparib-treated, Ga-GA@BSA-treated, and control groups (Figure 7D). Furthermore, immunohistochemical analysis was performed to verify the antitumor performance of the olaparib-Ga nanodrug in the xenograft tumor tissues. The expression of Ki-67, a cell proliferation marker, was notably downregulated in the olaparib-Ga group compared with the control, olaparib-treated, or Ga-GA@BSA-treated group (Figure 8A and B). Moreover, the expression of cleaved-caspase 3 protein was prominently upregulated following olaparib-Ga treatment, suggesting the improved therapeutic effect relative to olaparib or Ga-GA@BSA. Biosafety is a very important consideration for biomedical applications. The major organs, including the heart, lung, spleen, kidney, and liver, showed no detectable change in any of the four treatment groups (Figure 8C). In addition, the H&E staining of the heart, liver, spleen, lung, and kidney demonstrated that the mice treated with olaparib-Ga maintained normal tissue structure without distinct damage or inflammatory lesions (Figure 8D). Additionally, the blood results showed a small reduction in white blood cells, including neutrophils, in the olaparib-Ga-treated group (Figure 8E). Blood biochemistry results indicated no significant difference between the four groups, indicating good biocompatibility (Figure 8F).

Furthermore, the antitumor efficacy of the olaparib-Ga nanodrug against OVCAR3 tumors was also evaluated. Similarly, the ovarian cancer model was established by injecting OVCAR3-luc cells into mice intraperitoneally. The establishment of the OVCAR3-luc model was monitored using IVIS, and the antitumor efficacy of the four different treatments in mice bearing OVCAR3-luc tumors was examined. The detailed procedure for the animal experiments is depicted in Figure S8A. The bioluminescence images from each group (*n* = 5) and the quantification of the bioluminescent signal intensity at day 0 were similar (Figure S8B and C). However, the bioluminescence intensity of the olaparib-Ga group was the lowest among the four groups at day 22 (Figure S8B and C). The anticancer effect of the olaparib-Ga nanodrug in the OVCAR-3 cell-derived tumor model was similar to that of the SKOV3-derived tumor model. H&E staining of the heart, liver, spleen, lung, and kidney showed that the olaparib-Ga-treated group maintained a normal tissue structure feature without distinct damage or inflammatory lesions compared with the control, olaparib-treated, or Ga-GA@BSA-treated group (Figure S9A). Moreover, the routine blood and blood biochemistry results highlighted the biosafety of the olaparib-Ga nanodrug (Figure S9B and C). Altogether, the results from both models confirmed the good therapeutic effect and safety of olaparib-Ga, demonstrating that this nanodrug possesses clinical translational potential for ovarian cancer therapy.

### The Advantage of Olaparib-Ga Nanodrug *versus* Free Drugs

In order to uncover the advantage of the olaparib-Ga nanodrug in antitumor cell activity against HRR-proficient ovarian cancer cells, SKOV3 cells were treated with multiple sets of drugs including olaparib, Ga^3+^, gallic acid, olaparib plus Ga^3+^, olaparib plus gallic acid, gallic acid plus Ga^3+^, or olaparib-Ga nanodrug given at different drug doses. The cell variability was determined at 48 h after treatments. The sample concentration in each subgroup was calculated in correspondence with the olaparib concentration, and the concentration range was selected based on the IC_50_ of the olaparib-Ga nanodrug. As a result, all treatment groups exhibited dose-dependent tumor cell killing ability after 48 h treatments (Figure S10A). Of note, the olaparib-Ga nanodrug treatment groups showed significantly stronger antitumor activity when compared with the other treatment groups at concentrations of 66, 88, and 110 μM as calculated based on olaparib (Figure S10A). To further validate this finding, we have also chosen an 88 μM concentration and compared different treatment groups at 72 h. The results indicated that the olaparib-Ga nanodrug was still the most effective group in inhibiting SKOV3 cell viability (Figure S10B). In addition, colony formation assays with different treatment groups were conducted, and the same trend was detected as the cell variability assay (Figure S10C). Therefore, it is important to include olaparib-Ga in nanodrug formulations to achieve a more significant SKOV3 cell inhibition.

To further examine the *in vivo* antitumor advantages of the olaparib-Ga nanodrug over the free drugs, SKOV3-luc cells induced tumor bearing BALB/c nude mice were randomly divided into eight groups and then treated with the following different treatments: control, olaparib, Ga^3+^, gallic acid, olaparib plus Ga^3+^, olaparib plus gallic acid, gallic acid plus Ga^3+^, and olaparib-Ga nanodrug. During the treatment process, bioluminescence imaging was used to detect the SKOV3-luc cells’ signal intensity in tumors. The results demonstrated that the olaparib-Ga nanodrug treated tumor had the weakest signal intensity as compared to all the other treatment groups on day 22 (Figure S11A and B). However, the tumor signal intensity was not decreased statistically in the olaparib, Ga^3+^, gallic acid, olaparib plus Ga^3+^, olaparib plus gallic acid, or gallic acid plus Ga^3+^ treatment groups. The mice in all groups were then sacrificed, and the tumor tissues were collected for photographs and bioluminescence imaging. The general images of tumor tissues showed that the volume of tumors in the olaparib-Ga nanodrug treatment group was significantly smaller than that of the other groups (Figure S11C). Meanwhile, the tumor bioluminescence signal intensity in the olaparib-Ga nanodrug group was also the lowest as compared with the other groups (Figure S11D). Ki-67 IHC staining showed that the Ki-67 protein expression levels in the olaparib-Ga nanodrug treatment group were much lower than in the other treatment groups (Figure S11E). Thus, all the above results indicated that the olaparib-Ga nanodrug possessed the best superiority. Moreover, the preliminary biosafety was also evaluated. There was almost no significant difference in body weight among all the groups during the treatment process (Figure S12A). The blood routine and blood biochemistry markers did not change much for all eight groups (Figure S12B and C). The optical images of major organs including the heart, liver, spleen, lung, and kidney showed no detectable changes in all treatment groups (Figure S12D). The H&E staining of the heart, liver, spleen, lung, and kidney demonstrated that the mice in the eight groups possessed no obvious lethal damage (Figure S12E). Therefore, the above *in vitro* and *in vivo* studies demonstrated that designing the olaparib-Ga into a nanodrug formulation is necessary for effective treatment of HRR-proficient ovarian cancer.

## Conclusion

In this study, an “all-in-one” olaparib-Ga self-assembled nanodrug was designed for the treatment of HRR-proficient ovarian cancer, which exhibited an excellent suppressing capacity on the cell viability of two HRR-proficient ovarian cancer cell lines (SKOV3 and OVCAR3) compared with olaparib alone. Furthermore, the synergistic effect of gallium(III) and olaparib in HRR-proficient ovarian cancer cells was demonstrated. The enhanced therapeutic effect of olaparib-Ga may be attributable to suppression of RRM2 expression, activation of the Fe^2+^/ROS/MAPK pathway and HMOX1 signaling, inhibition of the PI3K/AKT pathway, and enhanced cleaved-caspase 3 and BAX protein expression. In addition, *in vivo* experiments involving SKOV3 and OVCAR3 cell-derived xenograft models demonstrated that the olaparib-Ga nanodrug could markedly inhibit tumor growth. Additionally, the olaparib-Ga nanodrug did not induce significant cytotoxicity *in vivo*. Therefore, we propose that the olaparib-Ga nanodrug possesses promising clinical application prospects for HRR-proficient ovarian cancer treatment.

## Experimental Methods

### Synthesis of Ultrasmall Olaparib-Ga Nanoparticles

Ultrasmall olaparib-Ga nanoparticles were prepared using a previous method.^32^ Specifically, 160 mg of BSA was added to 18 mL of deionized water and stirred for 0.5 h. A 10 mg/mL GaCl_3_ solution (2.2 mL) was then added to form a homogeneous solution. After stirring for 3 h, a 10 mg/mL gallic acid aqueous solution (2.05 mL) was dropped and continuously stirred at room temperature overnight. Subsequently, 100 μL of an olaparib dimethyl sulfoxide (DMSO) (Sigma) solution (100 mM) was introduced. After stirring for 6 h, olaparib-Ga nanoparticles were obtained by dialysis (molecular weight cutoff, 8000–14 000 Da) for 12 h. In all experiments, the concentration of the olaparib-Ga nanoparticles was determined using the Ga content.

### Cell Viability Assay and Apoptosis Assay

Olaparib powder was first immersed in DMSO to form the stock solutions (100 mmol/L) and stored at −80 °C for further use. For the cell viability assays, SKOV3 and OVCAR3 cells (5 × 10^3^ cells per well) were cultured into 96-well plates for 24 h. The culture medium was replaced with fresh DMSO or graded concentrations of olaparib and olaparib-Ga for 24, 48, and 72 h. Finally, the medium in each well was replaced with fresh culture medium including 10% Cell Counting Kit-8 solution (Dojindo Laboratories). The cells were cultured for another 2 h, and subsequently the absorbance in each well was read at a wavelength of 450 nm using a spectrophotometer (Thermo Fisher Scientific). Dose–response curves were generated using GraphPad Prism 9.0 (GraphPad Software, Inc.). Cell viability was examined relative to the control groups. Each assay was conducted in triplicate and repeated independently three times.

The cells were harvested, washed, and then resuspended in the binding buffer containing annexin V-fluorescein isothiocyanate (FITC) and propidium iodide (PI) (MultiSciences) for 15 min in the dark. The apoptotic rate was measured by flow cytometry (BD Biosciences, FACSVerse). Each assay was conducted independently three times.

### Colony Formation Assay

SKOV3 and OVCAR3 cells (1 × 10^3^) were seeded into six-well plates, then treated with the control, olaparib, or olaparib-Ga for 10 days. Once colonies had developed, they were fixed in 70% ethanol for 10 min and stained with 2% crystal violet solution (Sigma) for 5 min. Plates were then imaged using a camera (Canon). These experiments were repeated independently three times.

### RNA Extraction and RT-qPCR

The RNA extraction was conducted as previously described.^45^ The primers are listed in Table S2.

### Western Blot Analysis

SKOV3 and OVCAR3 cells were analyzed for the expression of RRM2 (Proteintech), γ-H2AX (Abcam), HMOX1 (Proteintech), PI3K (Cell Signaling Technology), p-PI3K (Cell Signaling Technology), AKT (Abcam), p-ATK (Cell Signaling Technology), BAX (Proteintech), cleaved-caspase 3 (Affinity), and β-actin (Proteintech) proteins, as previously described.^36^ The cells were collected and lysed after treatment (control, olaparib, or olaparib-Ga). Subsequently, the protein was transferred to PVDF membranes (Millipore), and Western blot analysis was performed with antibodies according to the manufacturer’s recommendations. The experiments were repeated independently three times.

### Immunofluorescence

Cells were fixed in 4% paraformaldehyde (Servicebio) and permeabilized with PBS-T (PBS, 0.3% Triton X-100). The cells were then blocked with 10% fetal bovine serum (FBS) containing 0.3% Triton X-100 (VETEC) for 1 h and then incubated with primary anti-γH2AX (Abcam) overnight. This was followed by staining with goat anti-mouse IgG (H+L) Alexa Fluor Plus-488 labeled secondary antibody (Invitrogen) for 1 h. Coverslips were mounted with a DAPI solution (Abcam). A confocal laser-scanning microscope (Olympus, FLUOVIEW FV1200) was used to visualize the fluorescence and acquire photos from four representative fields of each section.

### RNA Sequencing Library Construction and Sequencing

Total RNA was obtained from treated OVCAR3 cells using Trizol (Invitrogen). RNA quality was verified using a Bioanalyzer 2200 (Agilent). RNA samples with an RNA integrity number of >0.7 were used for cDNA library construction. RNA sequencing was performed by NovelBio Laboratory (Shanghai, China). Briefly, the complementary DNA (cDNA) libraries were constructed for each RNA sample using the TruSeq Stranded mRNA library prep kit (Illumina, Inc.) according to the manufacturer’s instructions. The libraries were quality controlled using an Agilent 2200 bioanalyzer and sequenced on a NovaSeq 6000 instrument using a 150-bp paired-end run.

### RNA Sequencing Mapping

Clean reads were obtained from the raw reads by removing the adaptor sequences and low-quality reads and then mapped to the human genome GRCh38 (NCBI) using HISAT2.^46^ HTseq was used to get gene counts. The RPKM method was used to determine the gene expression.^47^

### Intracellular Iron and ROS Evaluation

Intracellular iron content was investigated by using the fluorescent probe Phen Green SK (PGSK; Invitrogen) and FerroOrange (Dojindo Laboratories) according to the manufacturer’s instructions.. SKOV3 and OVCAR3 cancer cells were treated (control, olaparib, or olaparib-Ga) for 3 h, then incubated with 5 mM PGSK or 0.5 μg/mL FerroOrange at 37 °C for 30 min. For PGSK, the cells were then washed with PBS twice (washing was not necessary after FerroOrange treatment). Fluorescence images were recorded *via* a confocal laser scanning microscope (Olympus, Fluoview FV1200). The experiments were repeated independently three times.

Intracellular ROS generation was measured using a confocal laser scanning microscope (CLSM). SKOV3 and OVCAR3 cancer cells were cultured in CLSM culture dishes (2 × 10^5^ cells per well) overnight under normal culture conditions. The culture medium was then replaced with an equal volume of DMEM containing control, olaparib, or olaparib-Ga and incubated for 3 h. PBS containing 2.5 μM ROS was used to replace the medium, and the cells were then incubated at 37 °C for 30 min in the dark. The cells were then washed three times. Green fluorescence was detected to confirm intracellular ROS generation using a confocal laser scanning microscope (Olympus, Fluoview FV1200). The experiments were performed independently three times.

### *In Vivo* Tumor Xenograft Model

All the animal experiments including biodistribution and tumor xenograft model studies of olaparib-Ga were approved by the Institutional Animal Care and Use Committee (IACUC) of Zhejiang Chinese Medical University (no. IACUC-20200222-09). The studies were conducted in accordance with the National Institutes of Health Guide for the Care and Use of Laboratory Animals. Female, 5-week-old BALB/c nude mice were purchased from Shanghai Slack Laboratory Animal Center (Shanghai, China) and kept in a dedicated SPF facility in the animal research center of Zhejiang Chinese Medical University. For the xenograft experiments, approximately 2 × 10^6^ luciferase ovarian cancer cells (SKOV3-luc or OVCAR3-luc) were mixed in 100 μL of PBS, then injected intraperitoneally into the nude mice (*n* = 40). The SKOV3-luc or OVCAR3-luc tumor-bearing mice were used after 7 days. Subsequently, these mice were randomly divided into four groups (*n* = 5 per group): (a) intravenous injection of PBS; (b) intravenous injection of 200 μL of olaparib (0.5 mM); (c) intravenous injection of 200 μL of olaparib-Ga (0.5 mM with respect to olaparib); (d) intravenous injection with 200 μL of Ga-GA@BSA (the same amount with respect to the olaparib-Ga nanodrug). In the first 5 days, these treatments were repeated daily. Antitumor performance after treatment was monitored once per week using bioluminescence intensity measurements with the IVIS (PerkinElmer). The bioluminescence signal intensity of the tumors in each mouse was calculated using the Living Image software (PerkinElmer, USA). The results are shown as means + SD. Twenty-two days after different treatment processes, the blood sample of each mouse was first obtained and sent for routine blood and biochemistry analysis. Then, bioluminescence images was taken. Finally, the mice in different groups were sacrificed, and the major organs (heart, liver, spleen, lung, and kidney) of each group were isolated and placed in 4% paraformaldehyde for 24 h, then sectioned and H&E-stained for histological examination. Another 32 SKOV3-luc tumor-bearing mice used to determine the advantage of the olaparib-Ga nanodrug over free drugs were treated with control, olaparib, Ga^3+^, gallic acid, olaparib plus Ga^3+^, olaparib plus gallic acid, Ga^3+^ plus gallic acid, or olaparib-Ga (*n* = 4 each group) following a similar procedure to that mentioned above.

## Supporting Information Available

Methods including materials, characterization of nanodrug, drug loading and release, *in vivo* biodistribution study of olaparib-Ga, differentially expressed gene analysis, pathway analysis, and immunohistochemistry (PDF)

## References

[ref1] Siegel R. L.; Miller K. D.; Jemal A. Cancer Statistics, 2020. CA Cancer J. Clin.. 2020, 70 (1), 7–30. 10.3322/caac.21590. 31912902

[ref2] Li M.; Wang J.; Wang C.; Xia L.; Xu J.; Xie X.; Lu W. Microenvironment Remodeled by Tumor and Stromal Cells Elevates Fibroblast-Derived COL1A1 and Facilitates Ovarian Cancer Metastasis. Exp. Cell Res. 2020, 394 (1), 112153 10.1016/j.yexcr.2020.112153. 32589888

[ref3] Dai J.; Cheng Y.; Wu J.; Wang Q.; Wang W.; Yang J.; Zhao Z.; Lou X.; Xia F.; Wang S.; Tang B. Z. Modular Peptide Probe for Pre/Intra/Postoperative Therapeutic to Reduce Recurrence in Ovarian Cancer. ACS Nano 2020, 14 (11), 14698–14714. 10.1021/acsnano.9b09818. 33174739

[ref4] Moore K.; Colombo N.; Scambia G.; Kim B. G.; Oaknin A.; Friedlander M.; Lisyanskaya A.; Floquet A.; Leary A.; Sonke G. S.; Gourley C.; Banerjee S.; Oza A.; Gonzalez-Martin A.; Aghajanian C.; Bradley W.; Mathews C.; Liu J.; Lowe E. S.; Bloomfield R.; DiSilvestro P. Maintenance Olaparib in Patients with Newly Diagnosed Advanced Ovarian Cancer. N. Engl. J. Med. 2018, 379 (26), 2495–2505. 10.1056/NEJMoa1810858. 30345884

[ref5] Konstantinopoulos P. A.; Ceccaldi R.; Shapiro G. I.; D’Andrea A. D. Homologous Recombination Deficiency: Exploiting the Fundamental Vulnerability of Ovarian Cancer. Cancer Discovery 2015, 5 (11), 1137–1154. 10.1158/2159-8290.CD-15-0714. 26463832 PMC4631624

[ref6] Alsop K.; Fereday S.; Meldrum C.; deFazio A.; Emmanuel C.; George J.; Dobrovic A.; Birrer M. J.; Webb P. M.; Stewart C.; Friedlander M.; Fox S.; Bowtell D.; Mitchell G. BRCA Mutation Frequency and Patterns of Treatment Response in BRCA Mutation-Positive Women with Ovarian Cancer: a Report from the Australian Ovarian Cancer Study Group. J. Clin. Oncol. 2012, 30 (21), 2654–2663. 10.1200/JCO.2011.39.8545. 22711857 PMC3413277

[ref7] Hong T.; Lei G.; Chen X.; Li H.; Zhang X.; Wu N.; Zhao Y.; Zhang Y.; Wang J. PARP Inhibition Promotes Ferroptosis via Repressing SLC7A11 and Synergizes with Ferroptosis Inducers in BRCA-Proficient Ovarian Cancer. Redox Biol. 2021, 42, 101928 10.1016/j.redox.2021.101928. 33722571 PMC8113041

[ref8] Lord C. J.; Ashworth A. PARP inhibitors: Synthetic Lethality in the Clinic. Science 2017, 355 (6330), 1152–1158. 10.1126/science.aam7344. 28302823 PMC6175050

[ref9] Pommier Y.; O’Connor M. J.; de Bono J. Laying a Trap to Kill Cancer Cells: PARP Inhibitors and Their Mechanisms of Action. Sci. Transl. Med. 2016, 8 (362), 362ps17 10.1126/scitranslmed.aaf9246. 27797957

[ref10] Sonnenblick A.; de Azambuja E.; Azim H. A. Jr.; Piccart M. An Update on PARP Inhibitors-Moving to the Adjuvant Setting. Nat. Rev. Clin. Oncol. 2015, 12 (1), 27–41. 10.1038/nrclinonc.2014.163. 25286972

[ref11] Ashworth A. A Synthetic Lethal Therapeutic Approach: Poly(ADP) Rribose Polymerase Inhibitors for the Treatment of Cancers Deficient in DNA Double-Strand Break Repair. J. Clin. Oncol. 2008, 26 (22), 3785–3790. 10.1200/JCO.2008.16.0812. 18591545

[ref12] Audeh M. W.; Carmichael J.; Penson R. T.; Friedlander M.; Powell B.; Bell-McGuinn K. M.; Scott C.; Weitzel J. N.; Oaknin A.; Loman N.; Lu K.; Schmutzler R. K.; Matulonis U.; Wickens M.; Tutt A. Oral Poly(ADP-ribose) Polymerase Inhibitor Olaparib in Patients with BRCA1 or BRCA2Mutations and Recurrent Ovarian Cancer: a Proof-of-Concept Trial. Lancet 2010, 376 (9737), 245–251. 10.1016/S0140-6736(10)60893-8. 20609468

[ref13] Evers B.; Schut E.; van der Burg E.; Braumuller T. M.; Egan D. A.; Holstege H.; Edser P.; Adams D. J.; Wade-Martins R.; Bouwman P.; Jonkers J. A High-Throughput Pharmaceutical Screen Identifies Compounds with Specific Toxicity against BRCA2-Deficient Tumors. Clin. Cancer Res. 2010, 16 (1), 99–108. 10.1158/1078-0432.CCR-09-2434. 20008842 PMC2802735

[ref14] Chitambar C. R. Gallium-Containing Anticancer Compounds. Future Med. Chem. 2012, 4 (10), 1257–1272. 10.4155/fmc.12.69. 22800370 PMC3574811

[ref15] Chitambar C. R. Gallium Compounds as Antineoplastic Agents. Curr. Opin. Oncol. 2004, 16 (6), 547–552. 10.1097/01.cco.0000142071.22226.d2. 15627016

[ref16] Kircheva N.; Dudev T. Novel Insights into Gallium’s Mechanism of Therapeutic Action: A DFT/PCM Study of the Interaction between Ga(3+) and Ribonucleotide Reductase Substrates. J. Phys.Chem. B 2019, 123 (26), 5444–5451. 10.1021/acs.jpcb.9b03145. 31177779

[ref17] Kaneko Y.; Thoendel M.; Olakanmi O.; Britigan B. E.; Singh P. K. The Transition Metal Gallium Disrupts Pseudomonas aeruginosa Iron Metabolism and Has Antimicrobial and Antibiofilm Activity. J. Clin. Invest. 2007, 117 (4), 877–888. 10.1172/JCI30783. 17364024 PMC1810576

[ref18] Qi J.; Yao Q.; Qian K.; Tian L.; Cheng Z.; Wang Y. Gallium(III) Complexes of Alpha-N-heterocyclic Piperidylthiosemicarbazones: Synthesis, Structure-Activity Relationship, Cellular Uptake and Activation of Caspases-3/7/9. J. Inorg. Biochem. 2018, 186, 42–50. 10.1016/j.jinorgbio.2018.05.005. 29842999

[ref19] Aye Y.; Li M.; Long M. J.; Weiss R. S. Ribonucleotide Reductase and Cancer: Biological Mechanisms and Targeted Therapies. Oncogene 2015, 34 (16), 2011–2021. 10.1038/onc.2014.155. 24909171

[ref20] Bruijnincx P. C.; Sadler P. J. New Trends for Metal Complexes with Anticancer Activity. Curr. Opin..Chem. Biol. 2008, 12 (2), 197–206. 10.1016/j.cbpa.2007.11.013. 18155674 PMC2923029

[ref21] Desoize B. Metals and Metal Compounds in Cancer Treatment. Anticancer Res. 2004, 24 (3a), 1529–1544. 15274320

[ref22] Bonchi C.; Imperi F.; Minandri F.; Visca P.; Frangipani E. Repurposing of Gallium-Based Drugs for Antibacterial Therapy. Biofactor. 2014, 40 (3), 303–312. 10.1002/biof.1159. 24532037

[ref23] Krakoff I. H.; Newman R. A.; Goldberg R. S. Clinical Toxicologic and Pharmacologic Studies of Gallium Nitrate. Cancer 1979, 44 (5), 1722–1727. 10.1002/1097-0142(197911)44:5<1722::AID-CNCR2820440528>3.0.CO;2-C. 387208

[ref24] Mu X.; Yan C.; Tian Q.; Lin J.; Yang S. BSA-Assisted Synthesis of Ultrasmall Gallic Acid-Fe(III) Coordination Polymer Nanoparticles for Cancer Theranostics. Int. J. Nanomed. 2017, 12, 7207–7223. 10.2147/IJN.S146064. PMC5633299 29042770

[ref25] Yang W.; Guo W.; Le W.; Lv G.; Zhang F.; Shi L.; Wang X.; Wang J.; Wang S.; Chang J.; Zhang B. Albumin-Bioinspired Gd:CuS Nanotheranostic Agent for In Vivo Photoacoustic/Magnetic Resonance Imaging-Guided Tumor-Targeted Photothermal Therapy. ACS Nano 2016, 10 (11), 10245–10257. 10.1021/acsnano.6b05760. 27791364

[ref26] Ashrafizadeh M.; Zarrabi A.; Mirzaei S.; Hashemi F.; Samarghandian S.; Zabolian A.; Hushmandi K.; Ang H. L.; Sethi G.; Kumar A. P.; Ahn K. S.; Nabavi N.; Khan H.; Makvandi P.; Varma R. S. Gallic Acid for Cancer Therapy: Molecular Mechanisms and Boosting Efficacy by Nanoscopical Delivery. Food Chem. Toxicol. 2021, 157, 112576 10.1016/j.fct.2021.112576. 34571052

[ref27] Sakr T. M.; El-Hashash M. A.; El-Mohty A. A.; Essa B. M. (99m)Tc-Gallic-Gold Nanoparticles as A New Imaging Platform for Tumor Targeting. Appl. Radiat. Isot. 2020, 164, 109269 10.1016/j.apradiso.2020.109269. 32819507

[ref28] Zou Q.; Chang R.; Yan X. Self-Assembling Proteins for Design of Anticancer Nanodrugs. Chem. Asian. J. 2020, 15 (9), 1405–1419. 10.1002/asia.202000135. 32147947

[ref29] Sun H.; Chang R.; Zou Q.; Xing R.; Qi W.; Yan X. Supramolecular Protein Nanodrugs with Coordination- and Heating-Enhanced Photothermal Effects for Antitumor Therapy. Small 2019, 15 (52), e1905326 10.1002/smll.201970286. 31657116

[ref30] Li S.; Zou Q.; Li Y.; Yuan C.; Xing R.; Yan X. Smart Peptide-Based Supramolecular Photodynamic Metallo-Nanodrugs Designed by Multicomponent Coordination Self-Assembly. J. Am. Chem. Soc. 2018, 140 (34), 10794–10802. 10.1021/jacs.8b04912. 30102029

[ref31] Zhao F.; Shen G.; Chen C.; Xing R.; Zou Q.; Ma G.; Yan X. Nanoengineering of Stimuli-Responsive Protein-Based Biomimetic Protocells as Versatile Drug Delivery Tools. Chem.—Eur. J. 2014, 20 (23), 6880–6887. 10.1002/chem.201400348. 24828788

[ref32] An L.; Yan C.; Mu X.; Tao C.; Tian Q.; Lin J.; Yang S. Paclitaxel-Induced Ultrasmall Gallic Acid-Fe@BSA Self-Assembly with Enhanced MRI Performance and Tumor Accumulation for Cancer Theranostics. ACS Appl. Mater. Interfaces 2018, 10 (34), 28483–28493. 10.1021/acsami.8b10625. 30080382

[ref33] Catalan-Gomez S.; Redondo-Cubero A.; Palomares F. J.; Nucciarelli F.; Pau J. L. Tunable Plasmonic Resonance of Gallium Nanoparticles by Thermal Oxidation at Low Temperaturas. Nanotechnology. 2017, 28 (40), 405705 10.1088/1361-6528/aa8505. 28787277

[ref34] Huang H. H.; He C. L.; Wang H. S.; Mo X. M. Preparation of Core-Shell Biodegradable Microfibers for Long-Term Drug Delivery. J. Biomed Mater. Res. A 2009, 90 (4), 1243–1251. 10.1002/jbm.a.32543. 19572404

[ref35] Cao X.; Yu J.; Zhang Z.; Liu S. Bioactivity of Horseradish Peroxidase Entrapped in Silica Nanospheres. Biosens. Bioelectron. 2012, 35 (1), 101–107. 10.1016/j.bios.2012.02.027. 22410482

[ref36] Xu J.; Shen Y.; Wang C.; Tang S.; Hong S.; Lu W.; Xie X.; Cheng X. Arsenic Compound Sensitizes Homologous Recombination Proficient Ovarian Cancer to PARP Inhibitors. Cell Death. Discovery 2021, 7 (1), 259 10.1038/s41420-021-00638-2. 34552062 PMC8458481

[ref37] Hentze M. W.; Muckenthaler M. U.; Galy B.; Camaschella C. Two to Tango: Regulation of Mammalian Iron Metabolism. Cell 2010, 142 (1), 24–38. 10.1016/j.cell.2010.06.028. 20603012

[ref38] Goss C. H.; Kaneko Y.; Khuu L.; Anderson G. D.; Ravishankar S.; Aitken M. L.; Lechtzin N.; Zhou G.; Czyz D. M.; McLean K.; Olakanmi O.; Shuman H. A.; Teresi M.; Wilhelm E.; Caldwell E.; Salipante S. J.; Hornick D. B.; Siehnel R. J.; Becker L.; Britigan B. E.; Singh P. K. Gallium Disrupts Bacterial Iron Metabolism and Has Therapeutic Effects in Mice and Humans with Lung Infections. Sci. Transl. Med. 2018, 10 ( (460), ), 10.1126/scitranslmed.aat7520. PMC6637966 30257953

[ref39] Sandhu S. K.; Schelman W. R.; Wilding G.; Moreno V.; Baird R. D.; Miranda S.; Hylands L.; Riisnaes R.; Forster M.; Omlin A.; Kreischer N.; Thway K.; Gevensleben H.; Sun L.; Loughney J.; Chatterjee M.; Toniatti C.; Carpenter C. L.; Iannone R.; Kaye S. B.; de Bono J. S.; Wenham R. M. The Poly(ADP-ribose) Polymerase Inhibitor Niraparib (MK4827) in BRCA Mutation Carriers and Patients with Sporadic Cancer: A Phase 1 Dose-Escalation Trial. Lancet Oncol. 2013, 14 (9), 882–892. 10.1016/S1470-2045(13)70240-7. 23810788

[ref40] Yang M.; Chitambar C. R. Role of Oxidative Stress in the Induction of Metallothionein-2A and Heme Oxygenase-1 Gene Expression by the Antineoplastic Agent Gallium Nitrate in Human Lymphoma Cells. Free Radic. Biol. Med. 2008, 45 (6), 763–772. 10.1016/j.freeradbiomed.2008.05.031. 18586083 PMC2610863

[ref41] Yin H. Y.; Gao J. J.; Chen X.; Ma B.; Yang Z. S.; Tang J.; Wang B. W.; Chen T.; Wang C.; Gao S.; Zhang J. L. A Gallium(III) Complex that Engages Protein Disulfide Isomerase A3 (PDIA3) as an Anticancer Target. Angew. Chem., Int. Ed. 2020, 59 (45), 20147–20153. 10.1002/anie.202008432. 33448534

[ref42] Shigeta S.; Lui G. Y. L.; Shaw R.; Moser R.; Gurley K. E.; Durenberger G.; Rosati R.; Diaz R. L.; Ince T. A.; Swisher E. M.; Grandori C.; Kemp C. J. Targeting BET Proteins BRD2 and BRD3 in Combination with PI3K-AKT Inhibition as a Therapeutic Strategy for Ovarian Clear Cell Carcinoma. Mol. Cancer Ther. 2021, 20 (4), 691–703. 10.1158/1535-7163.MCT-20-0809. 33509905 PMC8026742

[ref43] Kircheva N.; Dudev T. Competition Between Abiogenic and Biogenic Metal Cations in Biological Systems: Mechanisms of Gallium’s Anticancer and Antibacterial Effect. J. Inorg. Biochem. 2021, 214, 111309 10.1016/j.jinorgbio.2020.111309. 33212396

[ref44] Zhan Y.; Jiang L.; Jin X.; Ying S.; Wu Z.; Wang L.; Yu W.; Tong J.; Zhang L.; Lou Y.; Qiu Y. Inhibiting RRM2 to Enhance the Anticancer Activity of Chemotherapy. Biomed.pharmacother. 2021, 133, 110996 10.1016/j.biopha.2020.110996. 33227712

[ref45] Lan H.; Yuan H.; Lin C. Sulforaphane Induces p53deficient SW480 Cell Apoptosis via the ROSMAPK Signaling Pathway. Mol. Med. Rep. 2017, 16 (5), 7796–7804. 10.3892/mmr.2017.7558. 28944886

[ref46] Kim D.; Langmead B.; Salzberg S. L. HISAT: A Fast Spliced Aligner with Low Memory Requirements. Nat. Methods 2015, 12 (4), 357–360. 10.1038/nmeth.3317. 25751142 PMC4655817

[ref47] Anders S.; Pyl P. T.; Huber W. HTSeq-A Python Framework to Work with High-Throughput Sequencing Data. Bioinformatics 2015, 31 (2), 166–169. 10.1093/bioinformatics/btu638. 25260700 PMC4287950

