# Supplementary material for: Breaking the Iron Homeostasis: A “Trojan Horse” Self-Assembled Nanodrug Sensitizes Homologous Recombination Proficient Ovarian Cancer Cells to PARP Inhibition
Source: ACS Nano. 2022 Aug 3;16(8):12786–800. doi: 10.1021/acsnano.2c04956 (PMC9413404; doi:10.1021/acsnano.2c04956)
Supplement: Supplementary file 1 — nn2c04956_si_001.pdf [file nn2c04956_si_001.pdf]

Supplementary Information

Breaking the Iron Homeostasis: A "Trojan Horse" Self-assembled Nanodrug Sensitizes Homologous Recombination Proficient Ovarian Cancer Cells to PARP Inhibition

Yangyang Li,[†,§] Yixuan Cen,[†,§] Yifeng Fang,[‡,§] Sangsang Tang,[†] Sen Li,[†] Yan Ren,[†] Hongbo Zhang,[||,¶,*] Weiguo Lu,[†,△,⊥,*] and Junfen Xu[†,△,*]

[†]Women's Reproductive Health Laboratory of Zhejiang Province, Women's Hospital, Zhejiang University School of Medicine, Hangzhou 310006, Zhejiang, China

[‡]Department of General Surgery, Sir Run Run Shaw Hospital, Zhejiang University School of Medicine, Hangzhou 310016, Zhejiang, China

[||]Pharmaceutical Sciences Laboratory, Åbo Akademi University, Turku FI-20520, Finland

[¶]Turku Bioscience Centre, University of Turku and Åbo Akademi University, Turku FI-20520, Finland

[△]Department of Gynecologic Oncology, Women's Hospital, Zhejiang University School of Medicine, Hangzhou 310006, Zhejiang, China

[⊥] Cancer Center, Zhejiang University, Hangzhou 310058, Zhejiang, China

[§] Y.L., Y.C. and Y.F. contributed equally to this work.

* Corresponding authors:

Correspondence to: Hongbo Zhang, email: hongbo.zhang@abo.fi; Weiguo Lu, email: lbwg@zju.edu.cn; Junfen Xu, email: xjfzu@zju.edu.cn

Supplementary Materials

Materials. BSA was purchased from Amresco (Solon, OH, USA). GA (99%) and Fe (III) chloride hexahydrate were purchased from Sinopharm Chemical Reagent (Shanghai, China). Dimethyl sulfoxide was purchased from sigma. Human HRR-proficient ovarian cancer cell lines OVCAR3 and SKOV3 were purchased from the American Type Cell Culture (ATCC). SKOV3 cells were grown in McCoy's 5A (BasalMedia, China) and OVCAR3 cells were cultured in Dulbecco's Modified Eagle's Medium (DMEM) (BasalMedia). The culture media contained 1% penicillin/streptomycin (Invitrogen) and 10% fetal bovine serum (FBS) (Gibco). Both cell lines were determined to be negative for mycoplasma and cultured at 37 °C with 5% CO_2. All reagents were used without further purification.

As the comparisons, Ga^{3+} ions (Ga^{3+}), gallic acid, Olaparib plus Ga^{3+} (Olaparib + Ga^{3+}), Olaparib plus gallic acid (Olaparib + Gallic acid), and gallic acid plus Ga^{3+} (Gallic acid + Ga^{3+}) were synthesized following the similar procedure of Olaparib-Ga nanoparticles, except replacing with equal volume of deionized water.

Characterization. The morphology and structure of Olaparib-Ga NPs were characterized by using a FEI Tecnai F20 transmission electron microscope (TEM). The phase structure of Olaparib-Ga NPs was determined by X-ray diffraction (XRD, X'Pert PRO MPD). The FTIR spectra of Olaparib, and Olaparib-Ga NPs were obtained via a PerkinElmer 580B infrared spectrophotometer on KBr pellets (Tensor 27, Bruker, Germany). The thermogravimetry (TG, DSCQ1000, AT, USA) curve of Olaparib-Ga. The hydrodynamic diameter of the Olaparib-Ga NPs was measured by a Zetasizer Nano-ZS (Malvern Instruments, UK). XPS measurement of Olaparib-Ga NPs was conducted h a VG ESCALAB MKII spectrometer (VG Scientific Ltd, UK). In all experiments, the Ga concentration within Olaparib-Ga NPs was defined by the ICP-MS (PerkinElmer NexION 300X).

Drug Loading and Release. A standard curve was obtained by measuring optical intensities of different concentrations of Olaparib (1, 2, 4, 6 and 8 μmoL) using a UV-2600 spectrophotometer (Shimadzu, Inc.) at characteristic wavelength of ~207 nm. The Olaparib drug loading efficiency was calculated by subtracting the original and remaining unreactive drug content. Specifically, the just resulting nanodrug solution were dialyzed against deionized water and unreactive Olaparib drug was collected. The Olaparib drug concentration was then determined. The same method was used to

calculate the concentration of gallic acid molecule and the characteristic absorption peak is ~264 nm. The centrifugation, drying and weighed experimental method was performed to obtain the concentration of the nanodrug. The concentration of the nanodrug was calculated as ~0.995 mg/mL. The mass ratio of Ga^{3+}: GA: BSA: Olaparib was 25.1: 62 : 10.2: 2.7 in final nanodrug.

The Olaparib releasing experiment was performed by immersing the dialysis bag (MW=15 kd) with 10 mL Olaparib-Ga nanodrug in 50 mL phosphate-buffered saline (PBS) solution of different pH values (7.4 and 5.8) under ~37 °C. At different time interval, 5 mL buffer solution was collected and replaced with an equal volume of fresh buffer solution. The released behavior of Olaparib drug was measured using an UV-2600 spectrophotometer. The Ga^{3+} releasing performance in PBS with different pH values was determined by inductively coupled plasma mass spectroscopy (ICP-MS, PerkinElmer NexION 300X)

***In vivo* biodistribution study of Olaparib-Ga.** We firstly synthesized the IR780-labeled Olaparib-Ga (IR780/Olaparib-Ga). Briefly, IR-780 dimethyl sulfoxide solution (0.1 mL; 1 mg/mL) was added to 10 mL of the Olaparib-Ga aqueous solution and stirred for 12 h at room temperature. The IR780/Olaparib-Ga were then dialyzed against deionized water overnight. For *in vivo* biodistribution experiment, 2×10^6 luciferase ovarian cancer SKOV3-luc cells were injected intraperitoneally into the BALB/c nude mice (n=6). The SKOV3-luc tumor-bearing mice were monitored using the Lumina LT IVIS (PerkinElmer, USA) after 7 days. Mice were then randomly divided into two groups, and intravenously injected with free IR780 (200μL) or IR780/Olaparib-Ga (200μL). The fluorescence signal of IR780 was measured using IVIS at 0 h (before injection) and 0.5 h, 1 h, 2 h, 6 h and 24 h after injection. At 6 h and 24 h post injection, mice were sacrificed under anesthesia. The tumors and vital organs including heart, liver, spleen, lung, kidney, and brain in each mouse were then isolated and imaged using IVIS.

Differentially expressed gene analysis. Differentially expressed gene analysis was applied utilizing DESeq2 algorithm under the following criteria: fold change > 1.5, or fold change < 0.67; P-value <0.05; FDR < 0.05.[1, 2]

Pathway analysis was used to find out the significant pathway of the differential genes according to KEGG database. We turn to the Fisher's exact test to select the significant pathway, and the threshold of significance was defined by P-value <0.05.[3]

Then, we picked the genes in enriched biological pathway and using Cytoscape for graphical representations of pathways.[4]

Immunohistochemistry (IHC). IHC was performed on paraffin-embedded ovarian tumor xenografts with the use of primary antibody to Ki-67 (Cell Signaling Technology) and cleaved Caspase 3 (Affinity) based on our previous study. All section slides were detected by standard hematoxylin and eosin (HE) staining.

Statistical analysis. All statistical analyses were performed using GraphPad Prism 9.0. All error bars used in this study are mean + s.d. of at the least three independent experiments. Statistically significant p values are indicated in figures and/or legends as *, $P < 0.05$; **$P < 0.01$, and ***$P < 0.001$.

References

(1) Love, M. I.; Huber, W.; Anders, S. Moderated estimation of fold change and dispersion for RNA-seq data with DESeq2. *Genome Biol* **2014,** *15* (12), 550.

(2) Benjamini, Y.; Drai, D.; Elmer, G.; Kafkafi, N.; Golani, I. Controlling the false discovery rate in behavior genetics research. *Behav Brain Res* **2001,** *125* (1-2), 279-84.

(3) Draghici, S.; Khatri, P.; Tarca, A. L.; Amin, K.; Done, A.; Voichita, C.; Georgescu, C.; Romero, R. A systems biology approach for pathway level analysis. *Genome Res* **2007,** *17* (10), 1537-45.

(4) Shannon, P.; Markiel, A.; Ozier, O.; Baliga, N. S.; Wang, J. T.; Ramage, D.; Amin, N.; Schwikowski, B.; Ideker, T. Cytoscape: a software environment for integrated models of biomolecular interaction networks. *Genome Res* **2003,** *13* (11), 2498-504.

Supplementary Tables and Figures

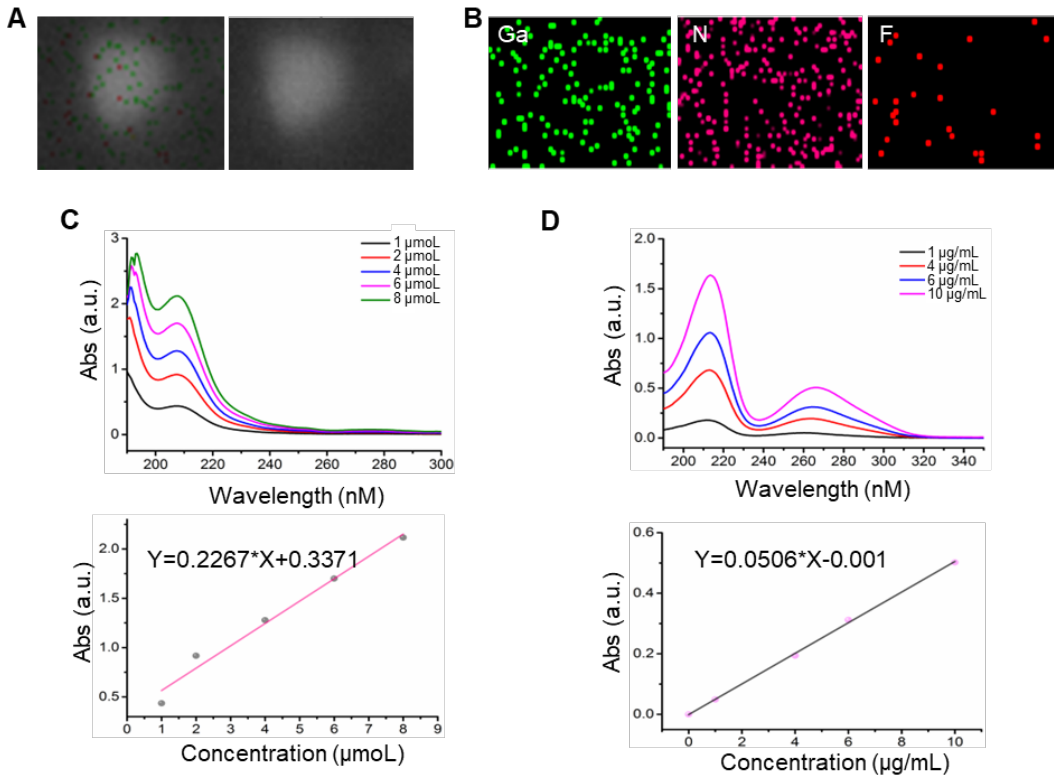

Figure S1 Features of Olaparib-Ga nanodrug. (a) STEM images and (b) elemental mapping patterns of Olaparib-Ga nanodrug. (c) The UV-vis absorption spectrum of Olaparib with different concentrations. The concentration fitting line of Olaparib is shown. (d) The UV-vis absorption spectrum of gallic acid molecule with different concentrations. The concentration fitting line of gallic acid molecule is shown.

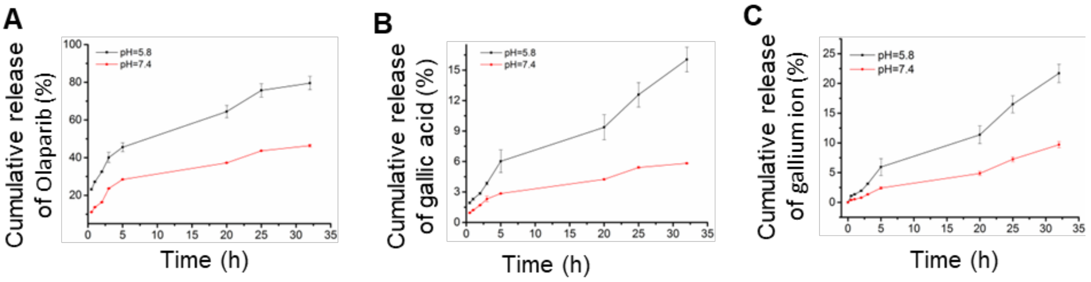

Figure S2. Drug releasing performance. (a) Cumulative Olaparib drug releasing behavior of Olaparib-Ga nanodrug in the different medium with two pH values. (b) Cumulative gallic acid releasing behavior of Olaparib-Ga nanodrug in the different medium with two pH values. (c) Ga^{3+} releasing performance in the two different medium.

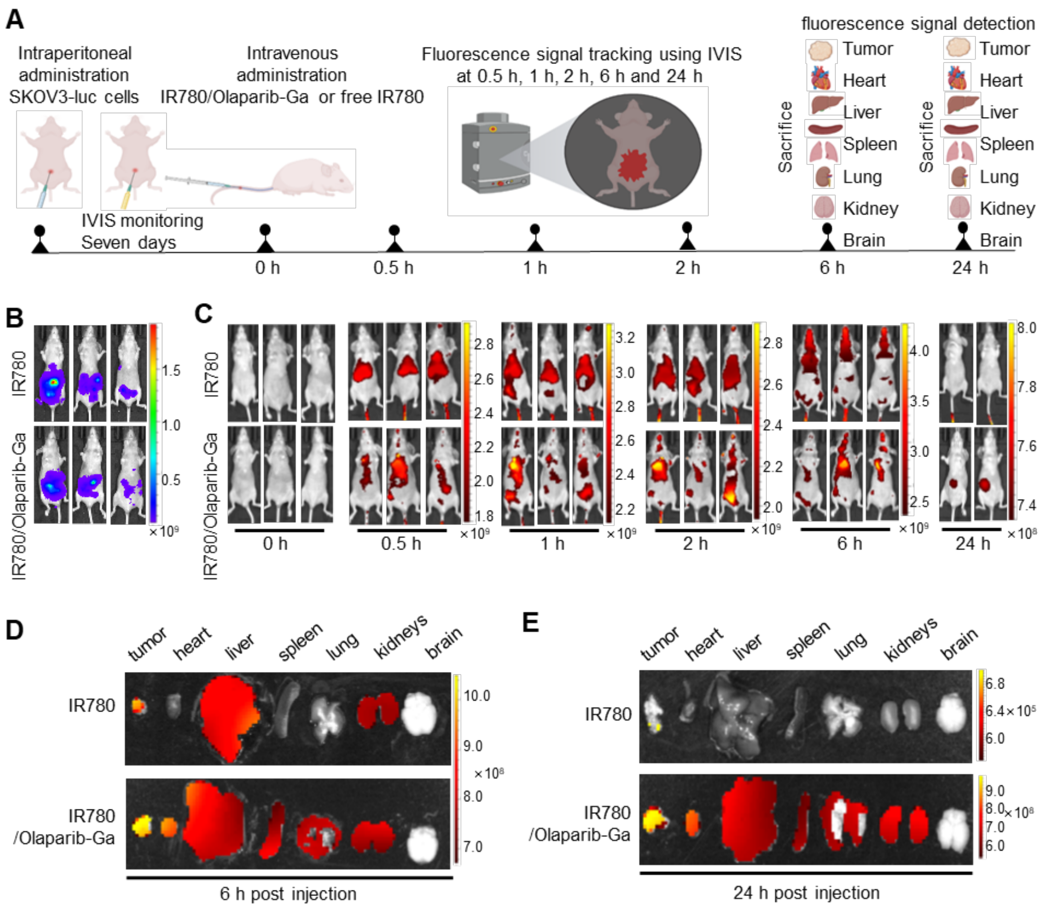

Figure S3. Biodistribution evaluation of free IR780 and IR780-Olaparib-Ga *in vivo*. (a) Schematic illustration of biodistribution study of IR780/Olaparib-Ga. (b) Bioluminescence images of mice before IR780/Olaparib-Ga or IR780 injection. (c) Fluorescence images of mice at 0, 0.5, 1, 2, 6 and 24 h post IR780/Olaparib-Ga or IR780 injection. (d) Fluorescence images of the xenograft tumor and major organs at 6 h post injection. (e) Representative fluorescence images of the xenograft tumor and major organs at 24 h post injection.

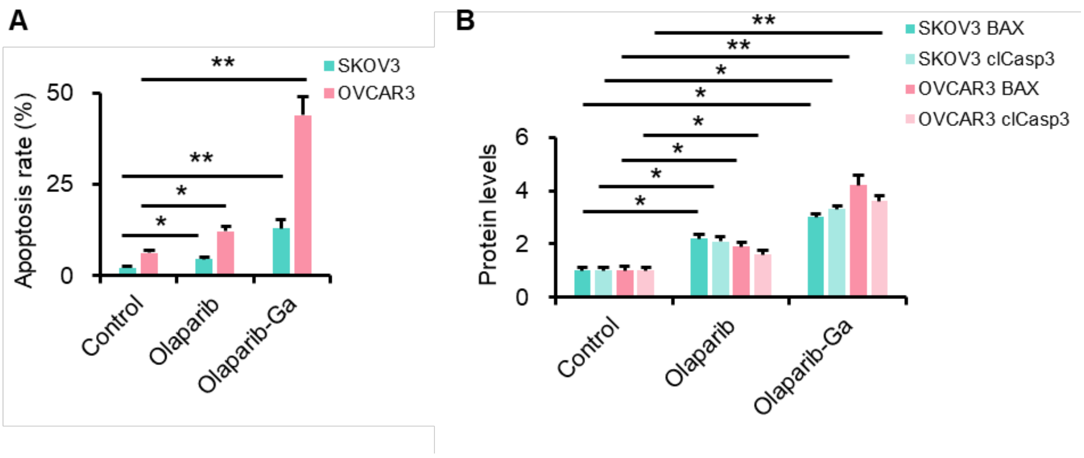

Figure S4. Olaparib-Ga nanodrug promotes apoptosis in SKOV3 and OVCAR3 cells. The SKOV3 and OVCAR3 cells were respectively treated with Olaparib or Olaparib-Ga nanodrug for 48 hours. The FACS analysis of cell apoptosis with Annexin V & PI staining was performed. The corresponding quantitative analysis of apoptotic rate was provided. *, $P < 0.05$; **, $P < 0.01$.

Table S1 Identification of 148 overlapped genes differentially expressed in both Olaparib and Olaparib-Ga groups.

Gene name	log2FC_Olaparib-Ga_vs_Control	Pvalue_Olaparib-Ga_vs_Control	log2FC_Olaparib_vs_Control	Pvalue_Olaparib_vs_Control
ABCC3	0.744776665	7.85163E-08	1.827369527	4.77314E-08
ABI3	-0.67165631	0.000113674	-0.648460594	8.19176E-06
ACOT2	-1.251832445	6.60956E-09	-0.609907758	0.004111696
ADAMTS6	-0.867223974	6.71164E-05	-0.846983404	3.49875E-06
ADAMTS9	1.56505111	2.09084E-08	1.795403603	5.70977E-13
ADGRG1	0.663763239	4.54926E-06	1.276867961	2.42389E-06
AGTRAP	0.761119978	1.39334E-08	0.742637433	5.01424E-11
ARG2	-1.45164165	1.33183E-09	-1.021394363	2.72326E-06
ARL14	-1.962562568	8.79977E-17	-0.99790669	4.04518E-08
ATF5	-0.842313082	6.06654E-12	-0.634153029	1.69977E-07
ATP8B3	-1.439375355	5.10126E-14	-0.706767519	1.60872E-05
BBC3	0.945592969	9.03503E-06	0.66214699	0.00466707
BHLHE40	1.252633849	0.000819767	0.996087218	0.000536437
BHLHE41	1.444443545	4.3749E-10	0.687203935	0.005639383
BIRC3	0.648891232	2.32955E-08	1.606870362	6.89097E-33
BMP2	1.205487702	5.43987E-06	0.793154868	0.001167752
C8orf46	-1.681581274	6.05183E-09	-1.389574118	2.02791E-08
CA9	-2.144322826	0.000526384	-3.315858507	1.84832E-09
CAPN6	-1.093761833	0.002680035	-1.848154333	8.73893E-07
CASC8	-0.772285376	0.00114923	-0.626015287	0.002570581
CD22	0.790196444	2.70295E-10	0.809583514	8.34861E-12
CDC20	-0.875457775	1.09858E-10	-0.620593949	6.30273E-19
CDC20P1	-0.933341658	0.000264078	-0.80234478	9.20115E-05
CDC42BPG	-1.093427235	0.002055161	-1.305385179	0.000129021
CDKN1A	2.403622144	6.95536E-54	0.877686025	2.68184E-08
CDKN2C	-1.031387791	1.36118E-08	-0.650648721	0.000020593
CENPE	-0.878049717	2.85669E-09	-0.586118981	3.25484E-11
CHRDL1	-1.294214401	7.03175E-05	-0.70877673	0.004948132
CLDN7	-1.549566215	4.5023E-08	-0.754343326	0.000526643
CLIC3	-1.826216943	2.57036E-06	-0.922425193	0.002584196
COL1A1	-0.679426465	0.000297757	-0.659868485	3.55931E-05
CREB3L1	-1.084556144	4.23345E-05	-0.735423619	0.001648515
CRYL1	0.607516829	0.001062394	0.721782131	3.04228E-05
CSF2	1.745076642	8.61827E-20	1.340078258	5.58751E-14
CTSD	0.638026616	1.67947E-11	0.64650401	5.68887E-13
CTSL	1.728599016	2.45736E-41	0.771640577	0.001155197
CYB561	-0.594270989	0.001107949	-0.646591714	6.3637E-06
CYGB	3.695009059	2.33969E-40	1.410229194	0.000331607
CYP1A1	3.706334666	1.7855E-63	3.953063646	5.76308E-25
CYP1B1	0.671864747	0.000432493	1.154469272	5.95638E-14
DIO2	-2.88824687	0.002279984	-1.785392419	7.01063E-05
DTX4	1.037426233	2.62338E-07	0.895395942	1.00843E-05
DYSF	1.028430913	1.40238E-15	0.96654323	2.10006E-15
E2F8	-0.714197549	2.56064E-07	-0.623413494	6.38673E-07
EFCAB13	1.552347975	5.69354E-10	0.872965349	0.000406766

EID3	-1.230795349	1.42659E-06	-0.67578603	0.002661294
ELF3	-1.245194777	0.000149175	-0.811708318	0.003783557
ESRP2	-0.855717202	1.10956E-08	-0.602275304	1.31245E-05
FAM107B	1.04213674	5.54109E-12	0.8333274	0.000418509
FAM129A	1.649309817	1.28665E-19	0.623217816	0.000322543
FAM83D	-0.659566012	7.62946E-12	-0.625847189	1.87169E-15
FTH1	1.910122044	2.98213E-67	0.707614435	2.19494E-15
FTH1P10	2.83524296	5.24985E-12	1.630341497	0.000254745
G0S2	1.019440421	0.00466569	1.452827961	0.001739878
GADD45B	1.746238096	0.00052432	0.621744322	3.50742E-10
GATA6	2.269294104	9.67655E-20	1.227731279	1.9511E-06
GBP1	1.623703711	2.17131E-08	1.077161273	0.00093132
GDF15	2.218707126	9.70121E-10	3.306022172	1.56179E-29
GJC2	-2.066366727	1.04505E-10	-1.089400549	0.00033592
GMFG	-0.737423484	0.000339927	-0.620122405	0.002803061
GOLGA7B	1.02151662	5.12525E-07	0.754492001	0.000508315
GPER1	-2.005251495	3.73085E-05	-1.086731915	0.004334895
GPR162	-1.156012509	3.46067E-05	-0.746851146	0.001809423
GYG2	-1.234680297	4.3103E-06	-0.718117362	0.001762324
H1F0	-1.407939988	1.68323E-45	-0.668425678	5.97433E-14
HERC5	1.126215764	2.8351E-07	0.76414598	0.002228604
HES1	0.969684202	2.47222E-07	0.936138586	2.08629E-11
HIST1H2BJ	-1.616440738	5.75406E-07	-0.837485852	0.001726723
HKDC1	0.877328326	6.48668E-10	0.701056886	2.83031E-07
HMOX1	6.742508857	1.381E-104	0.607615155	3.43255E-06
HNRNPR	-0.770887543	0.009645179	-0.753165268	0.005211763
IBTK	2.333940722	2.319E-09	1.345827157	0.002330227
ICK	-0.829393693	1.82352E-08	-0.736960381	2.05314E-09
IFI35	0.900222901	9.46832E-09	0.897105819	1.68407E-08
IFIH1	1.497273755	4.79626E-16	0.921593163	1.31154E-05
IFIT3	0.608483599	0.000647402	0.823946033	3.67499E-06
IGFBP6	-0.809772427	1.3733E-08	-0.751732958	4.81491E-09
IL24	3.691329228	1.10478E-11	4.342607557	1.61914E-18
INPP5J	-1.120097348	1.99972E-08	-0.652554864	0.000395228
ITGA11	0.920197901	6.59574E-06	2.322040845	5.86163E-42
ITGBL1	-2.516275383	1.07303E-25	-0.976702833	1.33209E-06
KIF20A	-1.313949407	0.000534328	-0.812147852	3.46013E-19
KLF4	0.956930686	2.84898E-06	0.724895902	0.000155065
KLHL24	1.343987053	3.03801E-06	0.875957466	0.000596571
LCP1	3.649018533	1.8022E-83	0.858562753	0.002267516
LCTL	-1.14021149	0.000129431	-1.163670218	3.74115E-05
LIF	0.719274401	3.83778E-10	1.00721151	1.27276E-24
LINC00467	0.635363616	0.005863934	0.665020689	0.001472146
LOC101927888	-2.027619736	0.000209703	-1.588087764	0.000930836
LOC102725231	0.819599646	0.000165033	0.597319334	0.003314877
LOC105378753	1.444193279	0.000368154	1.329180359	0.001167047
LOC344887	4.046421225	4.69606E-87	0.908782154	0.001489618
LOXL4	-1.644848637	5.5687E-12	-0.685374418	0.000475296
LPXN	1.487212457	8.85302E-16	0.770871887	1.38647E-05
LRIG1	0.700063664	0.000770287	0.742922071	9.06015E-05

MAP3K14	0.637624256	1.23055E-10	0.630727825	1.0695E-12
MAPK11	0.680582167	7.13615E-05	0.627061461	3.79076E-05
MPP4	-1.025270004	4.69394E-05	-1.13871783	7.3588E-07
MTSS1	1.313653375	1.38506E-13	1.477461956	5.3145E-21
NFKB2	1.190901225	2.09019E-39	0.632957208	2.11816E-11
NPTX1	1.913139268	3.55481E-12	4.555557418	6.3652E-121
NR2F2	-0.825275418	1.06126E-17	-0.620460536	1.84289E-13
NXNL2	-1.442895505	0.002411159	-1.359738373	0.003518403
NYNRIN	-1.611153877	9.28211E-09	-0.773518942	2.73006E-05
OLFML2A	-1.965221904	3.95461E-09	-1.001864654	0.000146066
PCDH1	1.43505547	7.51622E-11	0.64903329	0.001508609
PDE11A	-1.112606014	0.004753607	-0.908891193	0.004743291
PHF19	-0.981665352	2.37445E-22	-0.692290936	6.14398E-12
PI3	2.05080871	4.17049E-05	2.024301891	1.06726E-05
PLA2G4C	2.126962356	2.3354E-31	0.767743992	9.33061E-06
PLEKHF1	0.957758869	1.54581E-08	0.600684626	0.000452779
PLK1	-1.285576913	2.6925E-06	-0.733979084	1.13769E-23
PLXNA2	1.281194927	2.45728E-18	0.622244158	2.80534E-07
PPFIA4	-0.979380935	0.002502094	-0.955689638	0.000851105
PRCD	1.322852411	4.39042E-07	1.692644374	0.001986051
PTRH1	0.591454827	0.003123328	0.653923992	0.00019433
RASD2	0.935394599	0.013178049	1.115789187	0.000670774
ROM1	-0.743980624	0.012547714	-0.88249833	0.001516061
RRAGD	1.001175403	1.94812E-10	0.602660134	0.000290908
SAA1	1.078599321	2.60588E-15	0.787039794	1.4744E-14
SCG2	-2.919205301	1.36121E-11	-0.917468719	0.001318739
SDK2	1.126930274	4.97905E-05	0.803859999	0.003180565
SEL1L3	3.300781685	1.7093E-131	0.90107217	1.28516E-09
SERINC2	0.840397487	1.16534E-06	0.99337796	6.33491E-08
SH3KBP1	0.717512414	2.54338E-08	0.599717209	3.44852E-11
SLC16A6	2.14842251	1.8474E-22	2.620779717	9.79735E-50
SLC37A2	0.60390078	0.000466988	2.081178366	8.32604E-52
SLC3A2	1.152879873	0.000018161	0.815844171	0.000635094
SLC52A3	-0.901792231	0.001291695	-0.768569454	0.00228586
SLC7A11	2.086369656	5.25074E-49	1.042401006	0.003611807
SLCO1B7	-1.159322904	0.009932091	-1.785761802	8.03621E-05
SQRDL	1.807238806	9.36414E-18	0.83869041	0.001213102
SQSTM1	1.340516177	1.53E-25	0.820697737	5.0124E-20
TGFA	1.406860262	6.23222E-25	0.622712809	7.03164E-07
TIPARP	1.138869773	2.91498E-19	1.334551057	9.75569E-50
TMEM184A	-1.766547219	1.34815E-21	-0.597067584	5.50515E-05
TMEM71	-0.924928669	0.001305868	-0.939477752	0.000349382
TNC	-1.63380848	0.000216305	-0.993467899	1.18871E-08
TNFAIP3	0.680246287	1.61037E-08	0.87680205	2.56349E-12
TRAF1	1.509601357	4.8541E-27	0.944983824	4.85143E-09
TSPAN18	0.656636207	0.000119201	0.700477024	1.32552E-05
UNC13A	2.178014136	1.78966E-23	1.343223239	8.94514E-09
UPP1	1.932734	7.29689E-08	0.685247078	0.004613688
USP2	-1.322511779	0.000486594	-1.046656703	0.002175672
VAT1	0.801675227	6.6226E-16	0.767236715	1.08476E-19

VDR	0.595766227	8.02689E-05	1.073386306	1.17313E-12
VEGFA	1.184034754	7.94892E-17	0.904524767	2.84767E-12
ZMAT1	0.880674422	8.32616E-05	0.620253686	0.005054872

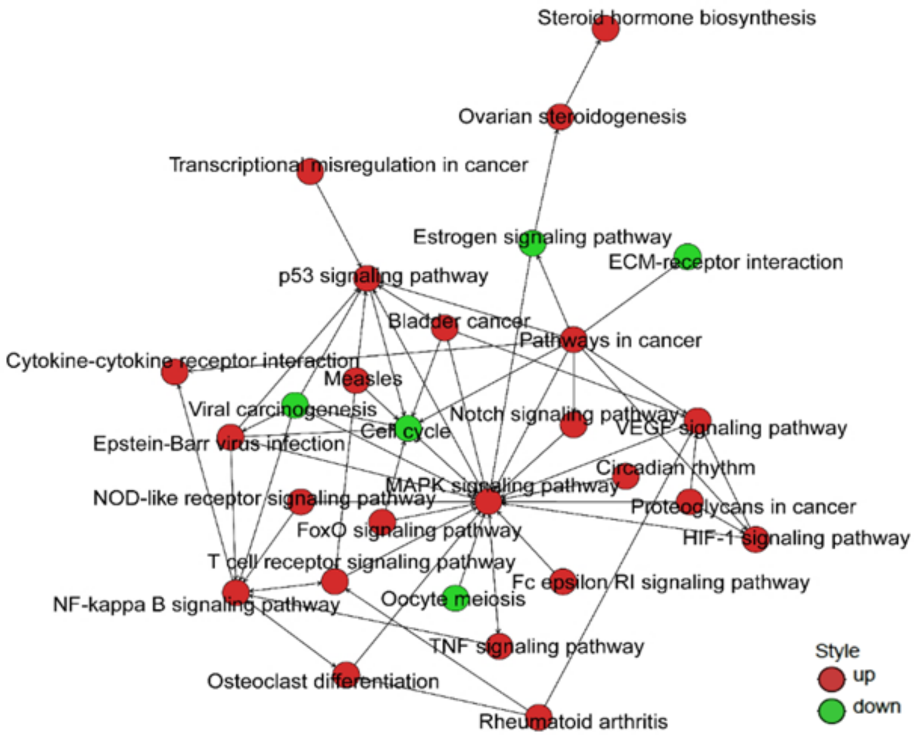

Figure S5. Path-Act-Network analysis of the 148 genes. 148 overlapped, differentially expressed genes crossing Control, Olaparib, and Olaparib-Ga nanodrug-treated groups were determined by RNA-seq. Path-Act-Network analysis was then performed. Red, activation of the pathways in Olaparib-treated and Olaparib-Ga-treated groups relative to the control group; Green, inhibition of the pathways.

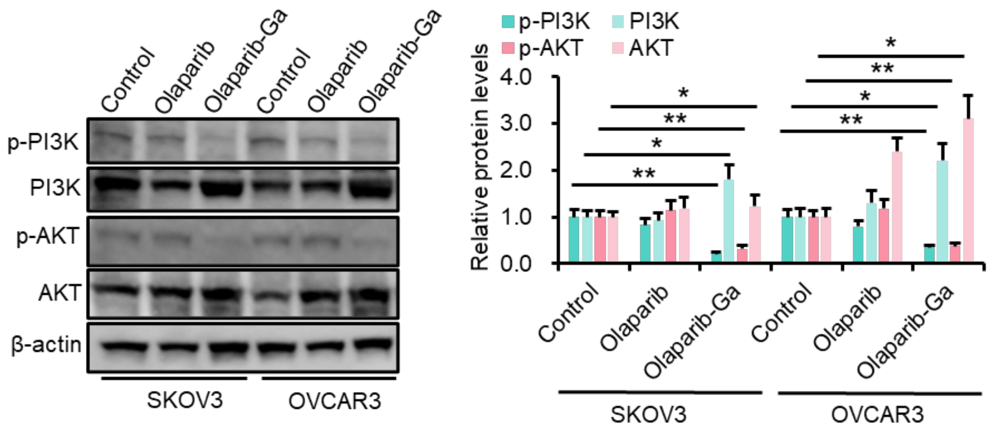

Figure S6. Olaparib-Ga nanodrug inhibits PI3K/AKT pathway in SKOV3 and OVCAR3cells. The SKOV3 and OVCAR3 cells were respective treated with Olaparib or Olaparib-Ga nanodrug for 48 hours. The protein levels of p-PI3K, p-AKT, PI3K and AKT were examined by Western blotting. The corresponding protein level quantitative analysis was provided on the right. *, $P < 0.05$; **, $P < 0.01$.

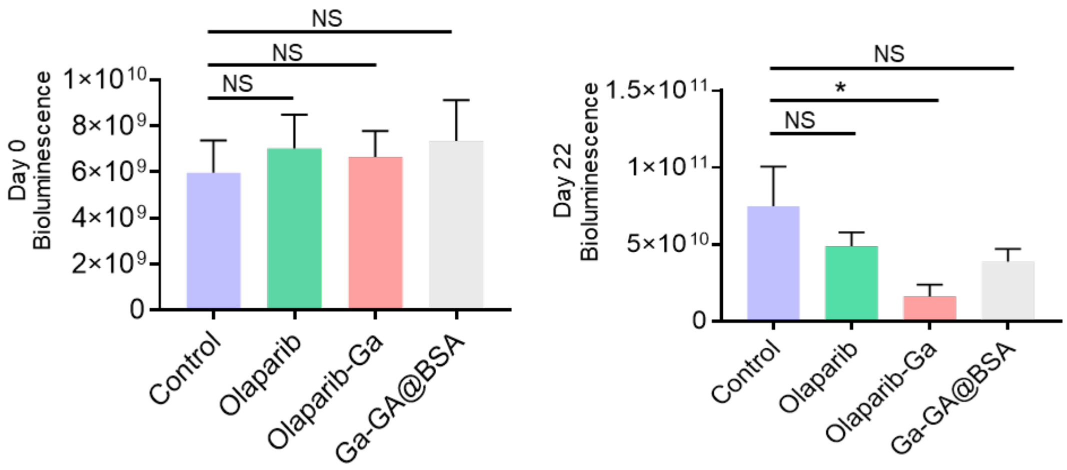

Figure S7. The anti-tumor effects of Olaparib-Ga nanodrug against the SKOV3-derived xenograft tumors. Tumor growth in mice bearing SKOV3-derived xenografts was measured and represented as mean + sd. *, $P < 0.05$; NS, not significant.

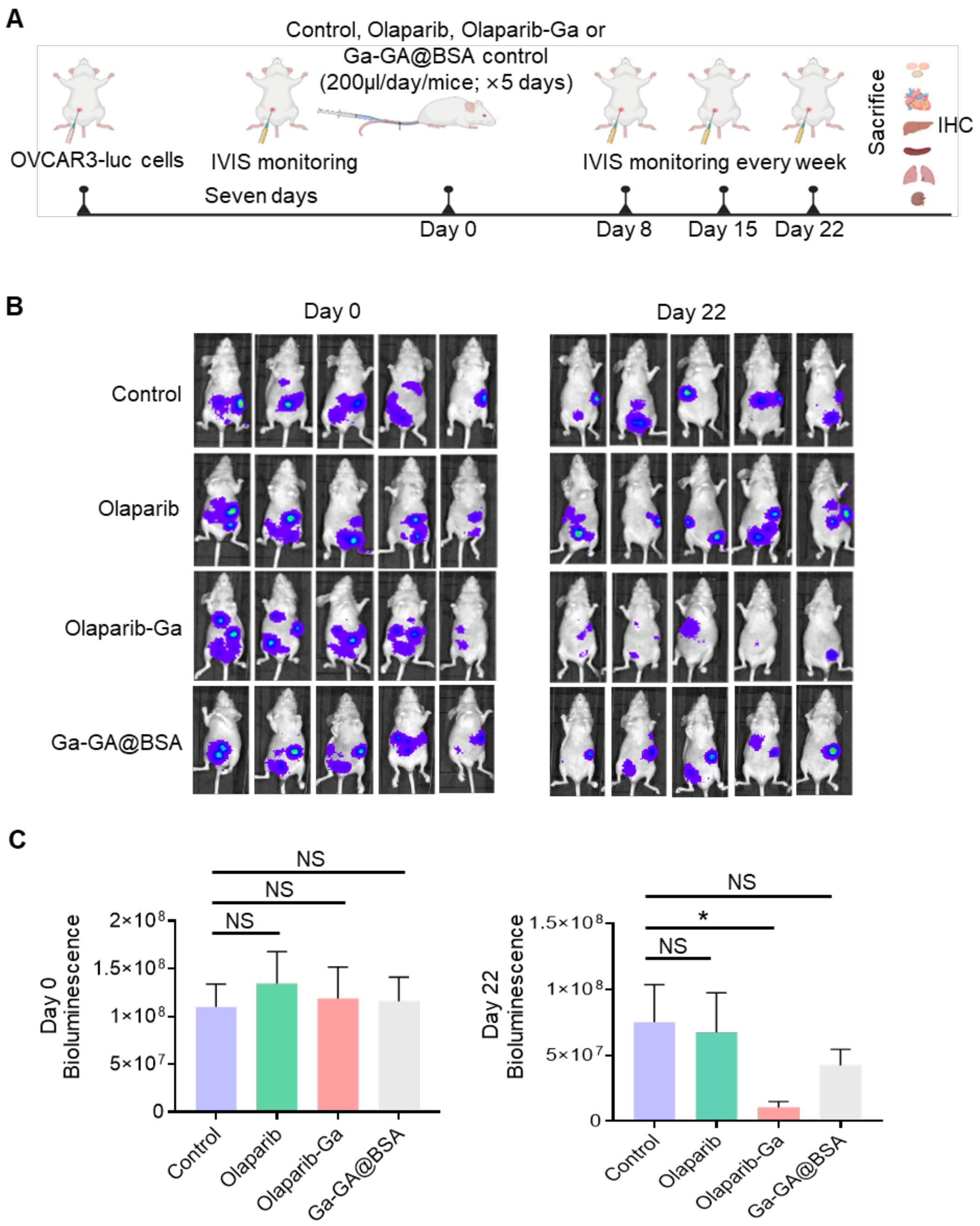

Figure S8. Anti-tumor efficacies of Olaparib-Ga nanodrug against OVCAR3 -derived animal model. (A) Experimental schematic of Olaparib-Ga nanodrug inhibiting the tumor growth in OVCAR3-luc-derived xenograft model. Mice bearing luciferase OVCAR3-derived tumors were treated with Olaparib or Olaparib-Ga or Ga-GA@BSA control (200 μL per mouse, QD × 5 days; n = 5 mice per group for each model). Tumor growth was monitored by bioluminescence imaging of the mice every week. (B) Representative bioluminescence images of mice bearing OVCAR3-derived xenograft tumors at day 0 and day 22. (C) Tumor growth in mice bearing OVCAR3-

derived xenografts were measured and represented as mean + sd. *, $P < 0.05$; NS, not significant.

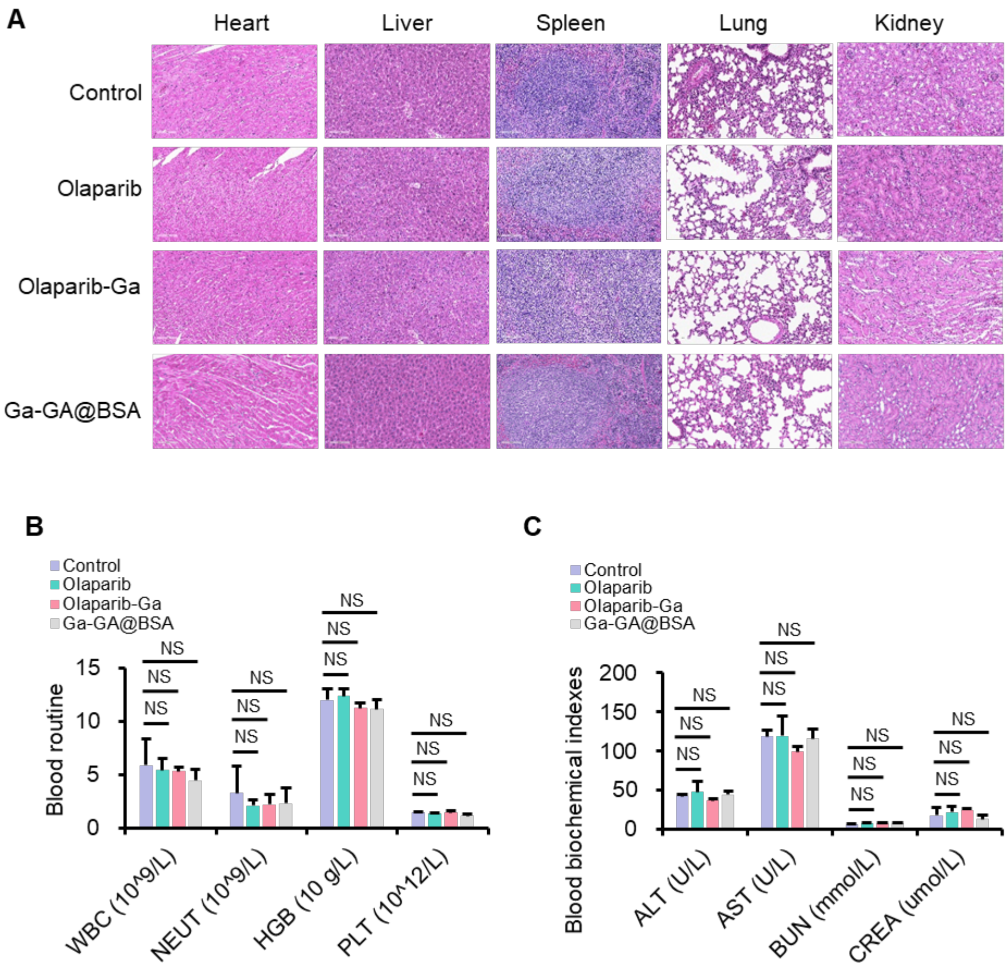

Figure S9. The preliminary toxicity analysis of Olaparib-Ga nanodrug in mice bearing OVCAR3-derived xenograft tumors. (A) Representative images of heart, liver, spleen, lung and kidney harvested from mice in each group. (B and C) Blood routine and blood biochemistry tests of the mice in each group were analyzed. Data are shown as mean + sd. NS, not significant.

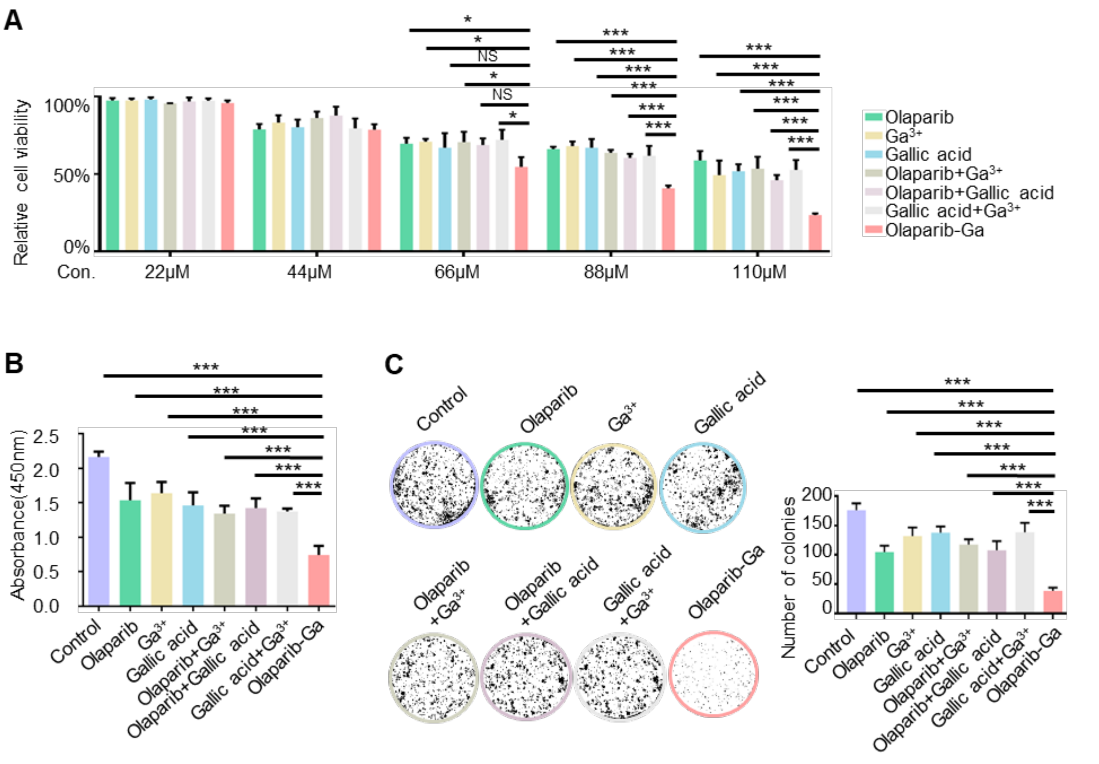

Figure S10. Cytotoxicity of Olaparib, Ga^{3+}, gallic acid, Olaparib plus Ga^{3+}, Olaparib plus gallic acid, Ga^{3+} plus gallic acid, or Olaparib-Ga in SKOV3 cells. (A) SKOV3 cells were treated with Olaparib, Ga^{3+}, gallic acid, Olaparib plus Ga^{3+}, Olaparib plus gallic acid, Ga^{3+} plus gallic acid, or Olaparib-Ga given at different drug doses. The sample concentration in each subgroup was calculated in corresponding to the Olaparib concentration, and the concentration range was selected based on the IC50 of the Olaparib-Ga nanodrug. The cell viability was detected at 48 h by CCK-8 assay. (B) SKOV3 cells were treated with Olaparib, Ga^{3+}, gallic acid, Olaparib plus Ga^{3+}, Olaparib plus gallic acid, Ga^{3+} plus gallic acid, or Olaparib-Ga given the drug concentration of 88 μM. The cell viability was analyzed at 72 h by CCK-8 assay. (C) SKOV3 cells were seeded for colony formation assay and treated continuously for 10 days with Olaparib, Ga^{3+}, gallic acid, Olaparib plus Ga^{3+}, Olaparib plus gallic acid, Ga^{3+} plus gallic acid, or Olaparib-Ga using the equal concentration with 10% of IC50 of Olaparib-Ga nanodrug. The data were represented as means + sd. * $P < 0.05$, *** $P < 0.001$. NS, not significant.

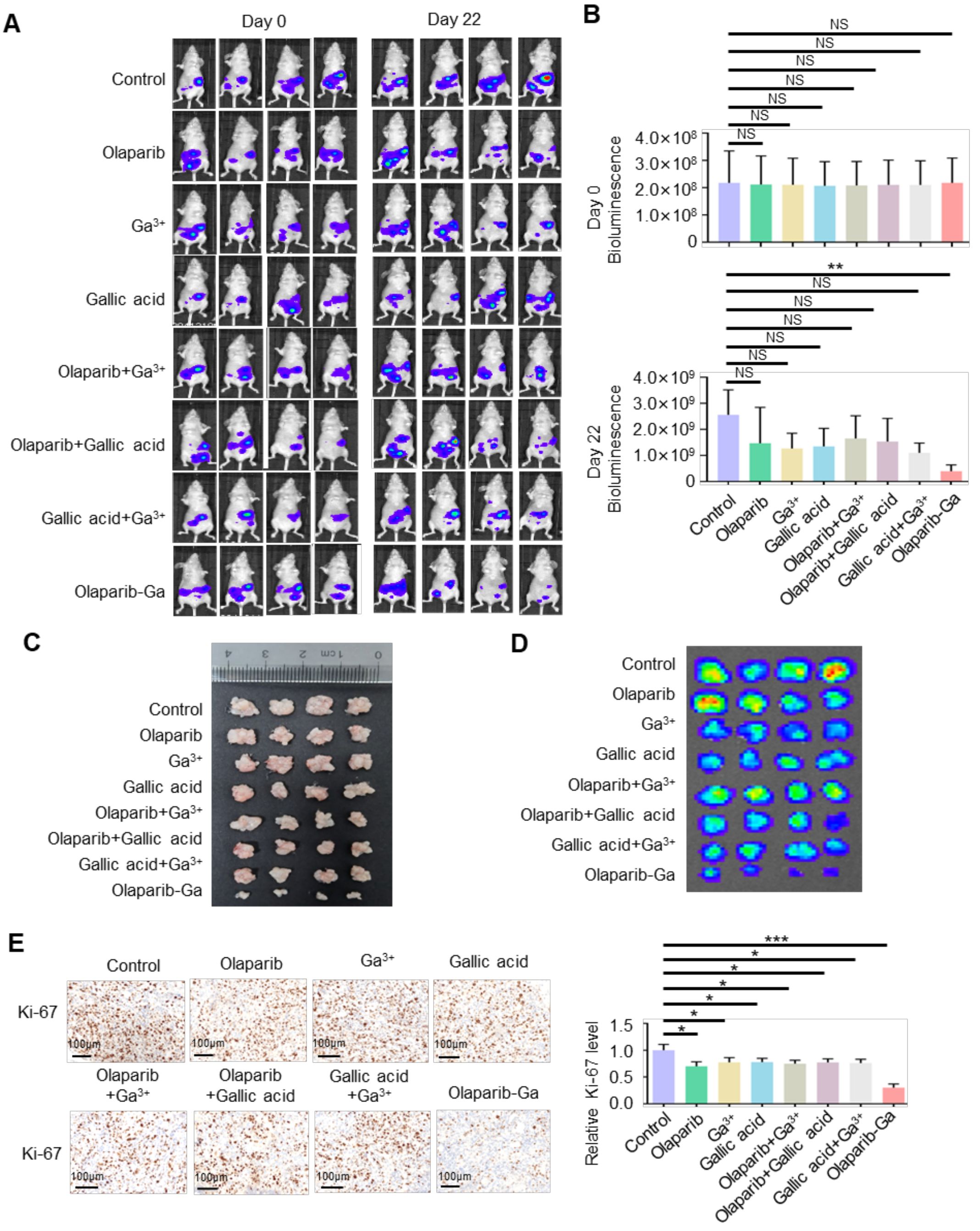

Figure S11. The anti-tumor efficacy of Olaparib, Ga^{3+}, gallic acid, Olaparib plus Ga^{3+}, Olaparib plus gallic acid, Ga^{3+} plus gallic acid, or Olaparib-Ga *in vivo*. (A) Bioluminescence images of SKOV3-luc tumor bearing mice before (Day 0, left panel) and post (Day 22, right panel) different drug treatments. (B) Bioluminescence signals of different groups in (A) were measured as represented. (C and D) Gross anatomy image (C) and bioluminescence image (D) of the dissected transplanted tumors in different groups. (E) Representative immunohistochemical staining analyses for Ki-67

protein of transplanted tumors in different groups. Scale bar, 100μm. The data were represented as means + sd. * $P < 0.05$, *** $P < 0.001$, NS, not significant.

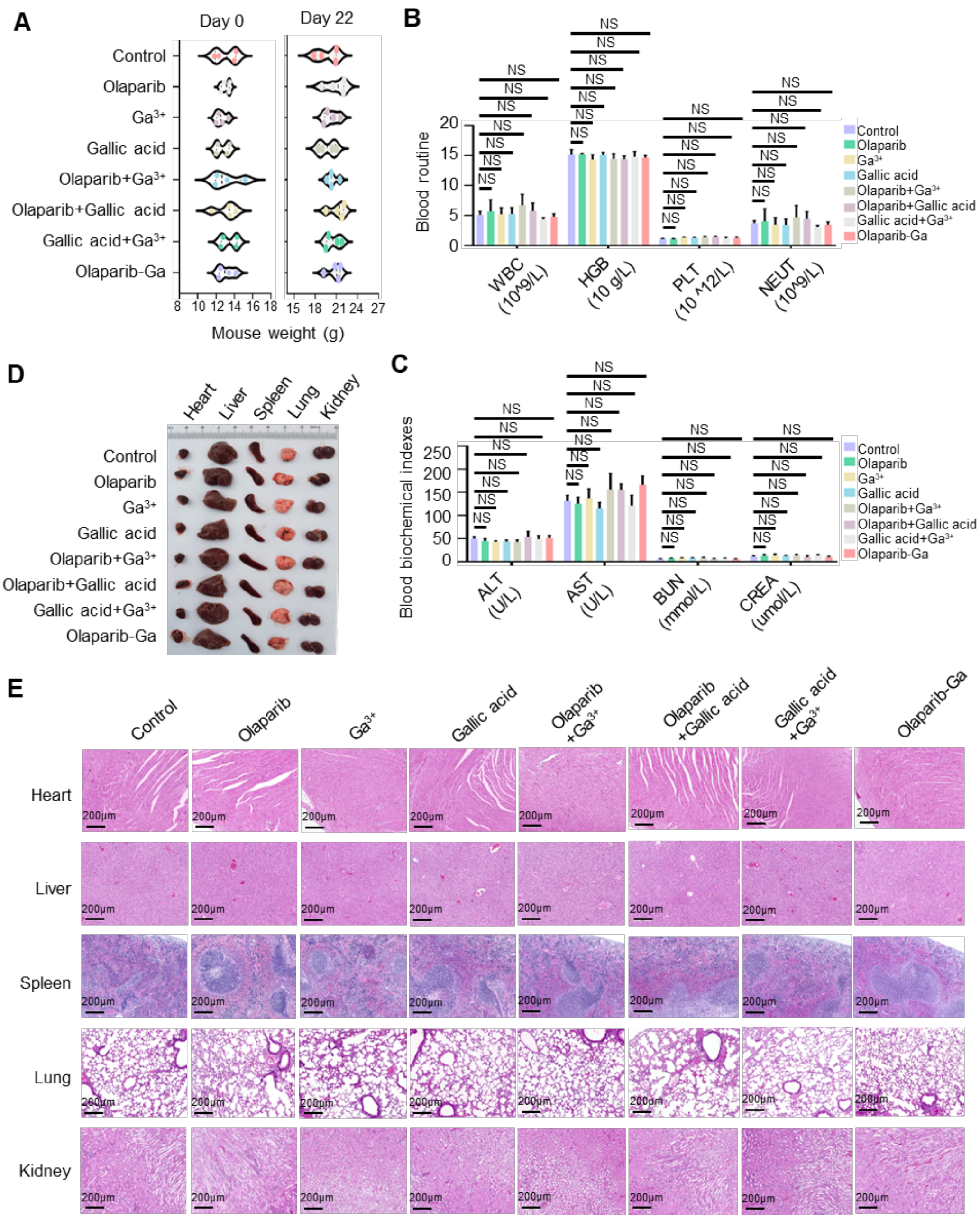

Figure S12. *In vivo* toxicology evaluation of Olaparib, Ga^{3+}, gallic acid, Olaparib plus Ga^{3+}, Olaparib plus gallic acid, Ga^{3+} plus gallic acid, or Olaparib-Ga in SKOV3-luc tumor bearing nude mice. (A) The body weights of SKOV3-luc tumor-bearing nude mice before (Day 0, left panel) and post (Day 22, right panel) different drug treatments. (B and C) Blood routine and blood biochemistry tests of nude mice in different groups. (D) Representative images of hearts, livers, spleens, lungs and kidneys of nude mice in different groups. (E) Representative H&E staining images of major organs in nude mice. Scale bar, 200μm. The data were represented as means + sd. NS, not significant.

Table S2 The primer sequences used for qRT-PCR in this study.

Primers	Strand	Sequences（5'-3'）
RRM2	Forward	CGGCGCGGGAGATTTAAAGG
	Reverse	CACGGAGGGAGAGCATAGTG
18s	Forward	TTAATTCCGATAACGAACGAGA
	Reverse	CGCTGAGCCAGTCAGTGTAG
CYGB	Forward	CCCGGCTCTATGCCAACTG
	Reverse	CCATGTGCTTGAACTGGCTGA
GADD45B	Forward	TACGAGTCGGCCAAGTTGATG
	Reverse	GGATGAGCGTGAAGTGGATTT
GATA6	Forward	CTCAGTTCCTACGCTTCGCAT
	Reverse	GTCGAGGTCAGTGAACAGCA
HMOX1	Forward	AAGACTGCGTTCCTGCTCAAC
	Reverse	AAGACTGCGTTCCTGCTCAAC
IBTK	Forward	ATGAGTTCACCCATGCCTGAC
	Reverse	TTTTCGCTCCCCTTTGTTACC
LCP1	Forward	GATCAGTGTCCGATGAGGAAATG
	Reverse	CCAGATCACCTGTAGCCATCA
NFKB2	Forward	ATGGAGAGTTGCTACAACCCA
	Reverse	CTGTTCCACGATCACCAGGTA
PLA2G4C	Forward	TGGATGCCGTCACGTACCT
	Reverse	CCAGAAGTCGGTCAGAGAGTA
SEL1L3	Forward	CTCTGCTACCTGAATGTTGTACC
	Reverse	CGGGAATGCTACTTCTGAACTC
SLC7A11	Forward	TCTCCAAAGGAGGTTACCTGC
	Reverse	AGACTCCCCTCAGTAAAGTGAC
TRAF1	Forward	TCCTGTGGAAGATCACCAATGT
	Reverse	GCAGGCACAACTTGTAGCC
DIO2	Forward	AGCAGACTACTGGTCTACTCAC
	Reverse	CACAGACTAATTTGCCTTGGGA
GJC2	Forward	GAGGTGCGACCGTTCTTTC
	Reverse	CTGACCACGTACATAACCAGC
GPER1	Forward	GCTCCGCATGATCCTCG
	Reverse	GACTGCTCGGTGCTGTCTG
ITGBL1	Forward	AGACCTACGACGGGAGCAC
	Reverse	ACCTGCATTAGAGCAGATGATGT
OLFML2A	Forward	CACGCCTACGTCCACAAGG
	Reverse	TCATAGTGCCTCAACTGCTCA
SCG2	Forward	ACCAGACCTCAGGTTGGAAAA
	Reverse	AAGTGGCTTTCATCGCCATTT
FOS	Forward	CCGGGGATAGCCTCTCTTACT
	Reverse	CCAGGTCCGTGCAGAAGTC
OSGIN1	Forward	CCCGGTCATCATTGTGGGTAA
	Reverse	GCTTCGTGTAGGGTGTGTAGC
ZFAND2A	Forward	GATCATTTTCCATACGCTGCAC
	Reverse	CGTCTGGTATCTGGCCCTTTT
DNAJA4	Forward	GGGATGTTTATGACCAAGGCG
	Reverse	GCCAATTTCTTCGTGACTCCA
TUSC3	Forward	GAGTTCCAGACGCTCAATCTTC

	Reverse	GCCAGGAGTTCGCCAGTATT
CLU	Forward	CCAATCAGGGAAGTAAGTACGTC
	Reverse	CTTGCGCTCTTCGTTTGTTTT
IL11	Forward	CGAGCGGACCTACTGTCCTA
	Reverse	GCCCAGTCAAGTGTCAGGTG
BMP4	Forward	ATGATTCCTGGTAACCGAATGC
	Reverse	CCCCGTCTCAGGTATCAAACT
GCLM	Forward	TGTCTTGGAATGCACTGTATCTC
	Reverse	CCCAGTAAGGCTGTAAATGCTC
ZSCAN31	Forward	AGAAGCCTCCCGACAA
	Reverse	GCCTTAGCCACTGATGAC
MLLT11	Forward	GGACCCTGTGAGTAGCCAGTA
	Reverse	CAGCTCCGACAGATCCAGT
PPP1R15A	Forward	ATGATGGCATGTATGGTGAGC
	Reverse	AACCTTGCAGTGTCCTTATCAG
GPRC5A	Forward	ATGGCTACAACAGTCCCTGAT
	Reverse	CCACCGTTTCTAGGACGATGC
SERPINA5	Forward	ATGCCCTTTTCACCGACCTG
	Reverse	TGCAGAGTCCCTAAAGTTGGTAG
SEMA3C	Forward	TTTGCGTGTTGGTTGGAGTAT
	Reverse	TCCTGTAGTCTAAAGGATGGTGG
PODXL	Forward	TCCCAGAATGCAACCCAGAC
	Reverse	GGTGAGTCACTGGATACACCAA
PLD1	Forward	GAGCCACGGGTAAATACCTCT
	Reverse	CCGCGTGTCCAGATTTTCTATG
PLAU	Forward	GGGAATGGTCACTTTTACCGAG
	Reverse	GGGCATGGTACGTTTGCTG
IL6	Forward	ACTCACCTCTTCAGAACGAATTG
	Reverse	CCATCTTTGGAAGGTTCAGGTTG
ID1	Forward	CTGCTCTACGACATGAACGG
	Reverse	GAAGGTCCCTGATGTAGTCGAT
ITGA2	Forward	CCTACAATGTTGGTCTCCCAGA
	Reverse	AGTAACCAGTTGCCTTTTGGATT
PDE4B	Forward	AACGCTGGAGGAATTAGACTGG
	Reverse	GCTCCCGGTTCAGCATTCT
DHRS3	Forward	ACTGAGTGCCATTACTTCATCTG
	Reverse	CATCACTGTCCATTAGGCTCTTC
ANGPT2	Forward	AACTTTCGGAAGAGCATGGAC
	Reverse	CGAGTCATCGTATTCGAGCGG
PTGS2	Forward	CTGGCGCTCAGCCATACAG
	Reverse	CGCACTTATACTGGTCAAATCCC
DKK1	Forward	CCTTGAACTCGGTTCTCAATTCC
	Reverse	CAATGGTCTGGTACTTATTCCCG
PTGES	Forward	TCCTAACCCTTTTGTCGCCTG
	Reverse	CGCTTCCCAGAGGATCTGC
CACNA1B	Forward	GACAACGTCGTCCGCAAATAC
	Reverse	CCCGATGAAATAGGGCTCCG
DDIT3	Forward	GGAAACAGAGTGGTCATTCCC
	Reverse	CTGCTTGAGCCGTTCATTCTC
FLNC	Forward	CTGGGCGATGAGACAGACG

	Reverse	GCGGATGGAACTTGCGGTA
FOSB	Forward	GCTGCAAGATCCCCTACGAAG
	Reverse	ACGAAGAAGTGTACGAAGGGTT
GADD45A	Forward	GAGAGCAGAAGACCGAAAGGA
	Reverse	CACAACACCACGTTATCGGG
GADD45G	Forward	CAGATCCATTTTACGCTGATCCA
	Reverse	TCCTCGCAAAACAGGCTGAG
HSPA6	Forward	CAAGGTGCGCGTATGCTAC
	Reverse	GCTCATTGATGATCCGCAACAC
PTPN7	Forward	GGGAGGTCACCCTACACTTTC
	Reverse	TGGTCTTGTATCGGTCCTTGG
PTPRR	Forward	ACCTATCGCCCATCACATTACA
	Reverse	GCGGTGGTAGCTTTGATCTCA
RASGRF1	Forward	TACTCGGCCATGTCACCCTT
	Reverse	GGGTCGTATCGCCCTCATC
